# The diagnostic and prognostic potential of extracellular vesicles as biomarkers for thyroid cancer: a systematic review

**DOI:** 10.1007/s00109-025-02594-1

**Published:** 2025-11-05

**Authors:** Nada Mabrouk Ahmed, Kevin Beatson, Jigisha Patel, Mohammad Mahmoud Rajab Eddama, Lucie H. Clapp, Tarek Abdel-Aziz

**Affiliations:** 1 Institute of Cardiovascular Sciences, University College London, London, UK; 2 Pathology Department, Alexandria University, Alexandria, Egypt; 3 Department of Surgical Biotechnology, Division of Surgery and Interventional Science, University College London, London, UK; 4 Endocrine Surgery Unit, University College London Hospitals, London, UK; 5 Department of Targeted Intervention, Division of Surgery and Interventional Science, University College London, London, UK

**Keywords:** Thyroid cancer, Extracellular vesicles, Exosomes, Biomarkers, Diagnosis, MiRNA

## Abstract

The incidence of thyroid cancer, the most common endocrine malignancy, has increased by 313% in the past four decades and is now the seventh most common cancer worldwide. There is an urgent need for non-invasive diagnostic, prognostic, and surveillance biomarkers to improve patient outcomes. Given the promising role of extracellular vesicles (EVs) as liquid biopsy biomarkers, a systematic review of the literature was conducted to evaluate their diagnostic and prognostic utility in thyroid cancer. We also assessed the quality of included studies giving special emphasis to methodology, reporting standards, and adherence to MISEV2018 guidelines. A comprehensive search was conducted across Web of Science, Ovid Medline, Ovid Embase, Scopus, and Emcare for English-language studies published from inception to March 2025. Search terms included a combination of relevant keywords and subject Headings. A total of 40 studies met the inclusion criteria. Most focused on papillary thyroid carcinoma, with relatively a minority investigating follicular thyroid carcinoma. The majority examined small EVs (exosomes), with microRNAs (miRNAs) being the most studied EV biomarkers, followed by proteins, circular RNAs, long non-coding RNAs, mRNAs, DNA, procoagulant phospholipids, and biophysical characteristics. No studies investigated EV lipids or metabolites as potential thyroid cancer biomarkers. This systematic review highlights the strong potential of EVs as diagnostic and prognostic biomarkers in thyroid cancer. However, larger prospective patient cohorts are needed to validate current findings. Clinical translation will require standardised methodologies and robust comprehensive reporting aligned with MISEV2018 guidelines, enhancing reproducibility and paving the way for multicentre clinical trials.

## Introduction

**Fig. 1:**
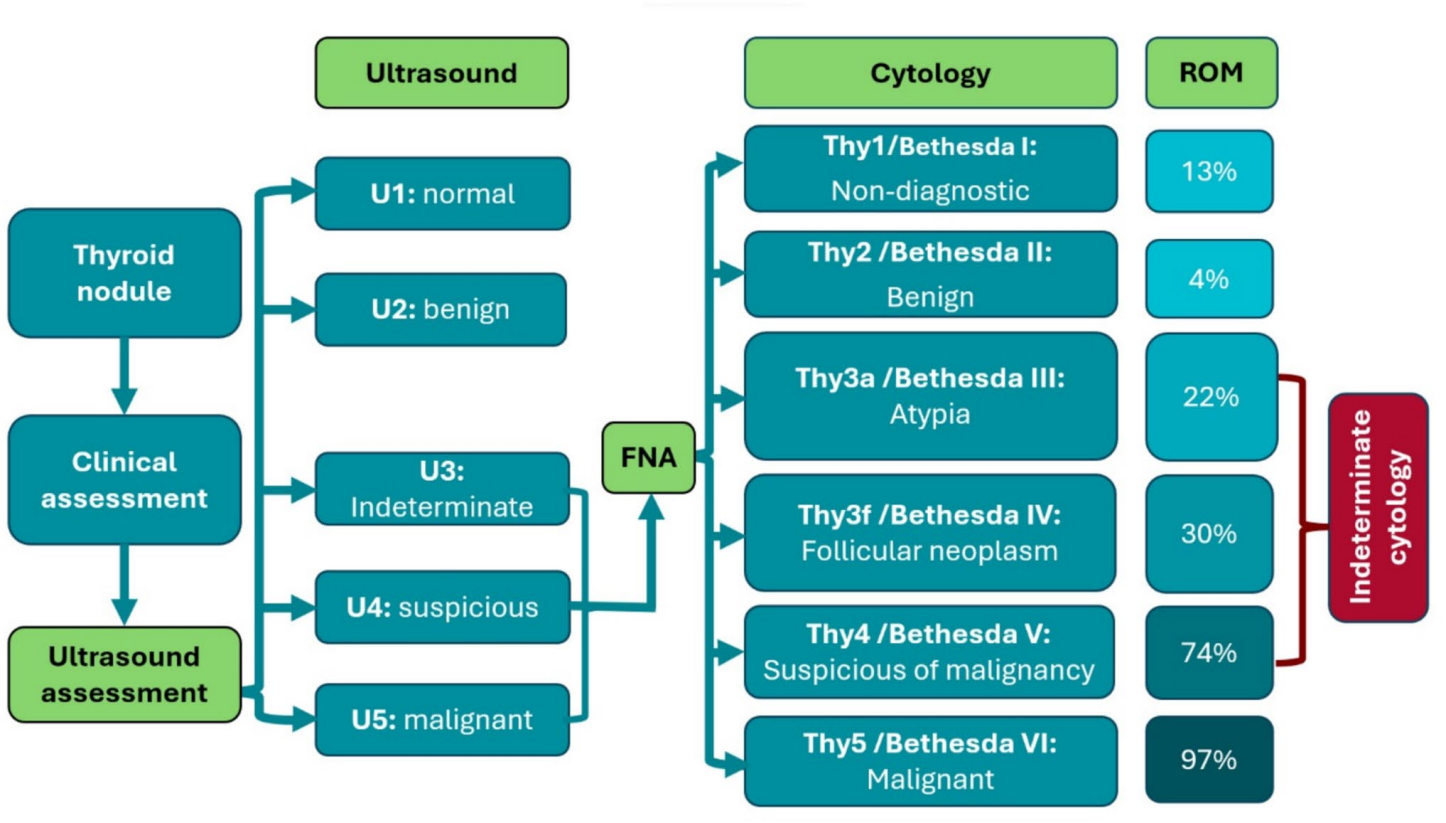
Summary of the clinical diagnostic pathway for thyroid nodules. After clinical assessment, patients undergo an ultrasound scan. Nodules with suspicious ultrasound features U3-U5 are subjected to fine needle aspiration (FNA) cytology (FNAC). Each cytological category is associated with a risk of malignancy (ROM), as defined by the Royal College of Pathologists in the UK and/or the 2023 Bethesda System for Reporting Thyroid Cytopathology [5]

Thyroid cancer (TC) is the commonest endocrine malignancy. Incidence has increased 313% over four decades. TC is the seventh most common cancer worldwide and fifth most common in women [1, 2]. Thyroid nodules (TNs) are common, with a prevalence of palpable TNs being 5% in females and 1% in males. Detection with high-resolution ultrasound increases this to around 45% in females and 30% in males [3]. Amongst TNs, 7–15% are malignant, based on ultrasound and fine needle aspiration cytology (FNAC) [4] (Fig. 1).

**Fig. 2:**
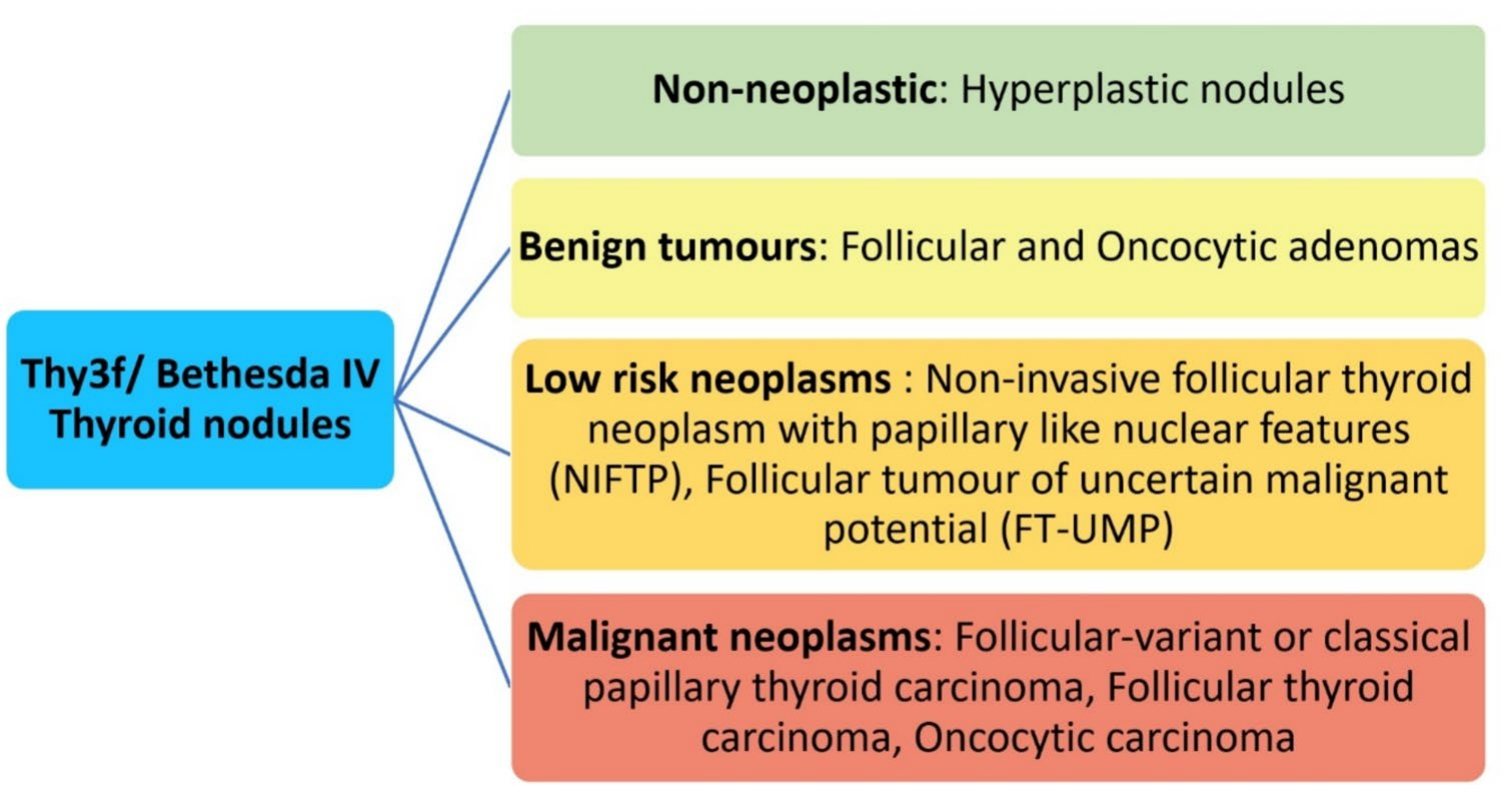
Final histopathological differential diagnoses for indeterminate follicular thyroid nodules (Thy3F/Bethesda IV)

Although FNAC can diagnose papillary TC (PTC) with high sensitivity and specificity [6, 7]. Up to 16% of samples are inadequate for evaluation, and approximately 8% of these are cancerous [8]. Furthermore, around 20–40% of TNs remain indeterminate even after adequate FNAC [9, 10]. These include follicular TNs, which have a wide range of differential diagnoses, including non-neoplastic hyperplastic nodules, benign follicular adenomas (FAs), malignant follicular TC (FTC), follicular variant of PTC (FV-PTC), and the low-risk neoplasms non-invasive follicular thyroid neoplasm with papillary-like nuclear features (NIFTP) [7] (Fig. 2). Follicular TNs mostly require diagnostic surgery to rule out malignancy [4]. This results in numerous unindicated, potentially avoidable surgical interventions, as the cancer rate at final histology is only < 30% [11]. Moreover, diagnostic Hemithyroidectomy carries the risk of surgical complications, and approximately 30% of patients require lifelong thyroid hormone replacement [12], impacting long-term quality of life [12].

Lymph node metastasis (LNM) or distant metastasis occurs in 20–50% of patients with PTC and 6–20% of patients with FTC, respectively [4, 13]. Metastasis is associated with recurrence, poorer prognosis, and worse overall survival in TC patients [4]. TC tends to spread initially to the neck’s central compartment lymph nodes, where ultrasound only has 30% sensitivity in detecting LNM (compared to 70% sensitivity for the lateral compartment) [14].

Monitoring of TC recurrence after treatment remains a clinical challenge. Serum thyroglobulin is the current standard biomarker for surveillance in differentiated thyroid cancer (DTC), with rising levels during follow-up suggesting potential recurrence, warranting further investigation [4]. However, its sensitivity is significantly reduced in patients who have undergone lobectomy alone, without completion thyroidectomy. This is because the remaining lobe of the thyroid continues to produce thyroglobulin, making it difficult to distinguish between benign residual production and cancer recurrence [15]. Furthermore, 25% of DTC patients have anti-thyroglobulin antibodies which interfere with thyroglobulin measurement, further limiting the reliability of this test [16].

The limitations of current gold standard investigations underscore a need for novel biomarkers to improve the diagnosis, prognosis, preoperative staging, and postoperative surveillance of TC. The discovery of novel biomarkers may preclude the need for diagnostic surgery, lower the risk of treatment-related morbidity, and alleviate burdens on resource-limited healthcare systems, while also elucidating potential therapeutic targets.

Liquid biopsy is a non-invasive technique involving analysis of molecular alterations from tumour-derived components within body fluids. Its utilities include early diagnosis, prognosis, and monitoring of response to therapy or relapse [17]. Amongst the liquid biopsy-based components showing great promise as biomarkers are extracellular vesicles (EVs) [18]. EVs are anucleate nanosized vesicles formed of a cytosolic centre bound by a phospholipid bilayer membrane and play major roles in intercellular communication by transferring their contents [19]. They are shed from all cell types, including tumour cells, into most body fluids [20, 21]. They express membrane receptors and contain bioactive cargo, including RNA, DNA, and proteins that originate from their parent cell. Thus, EVs may provide information of the disease state of their cell of origin [22].

**Fig. 3:**
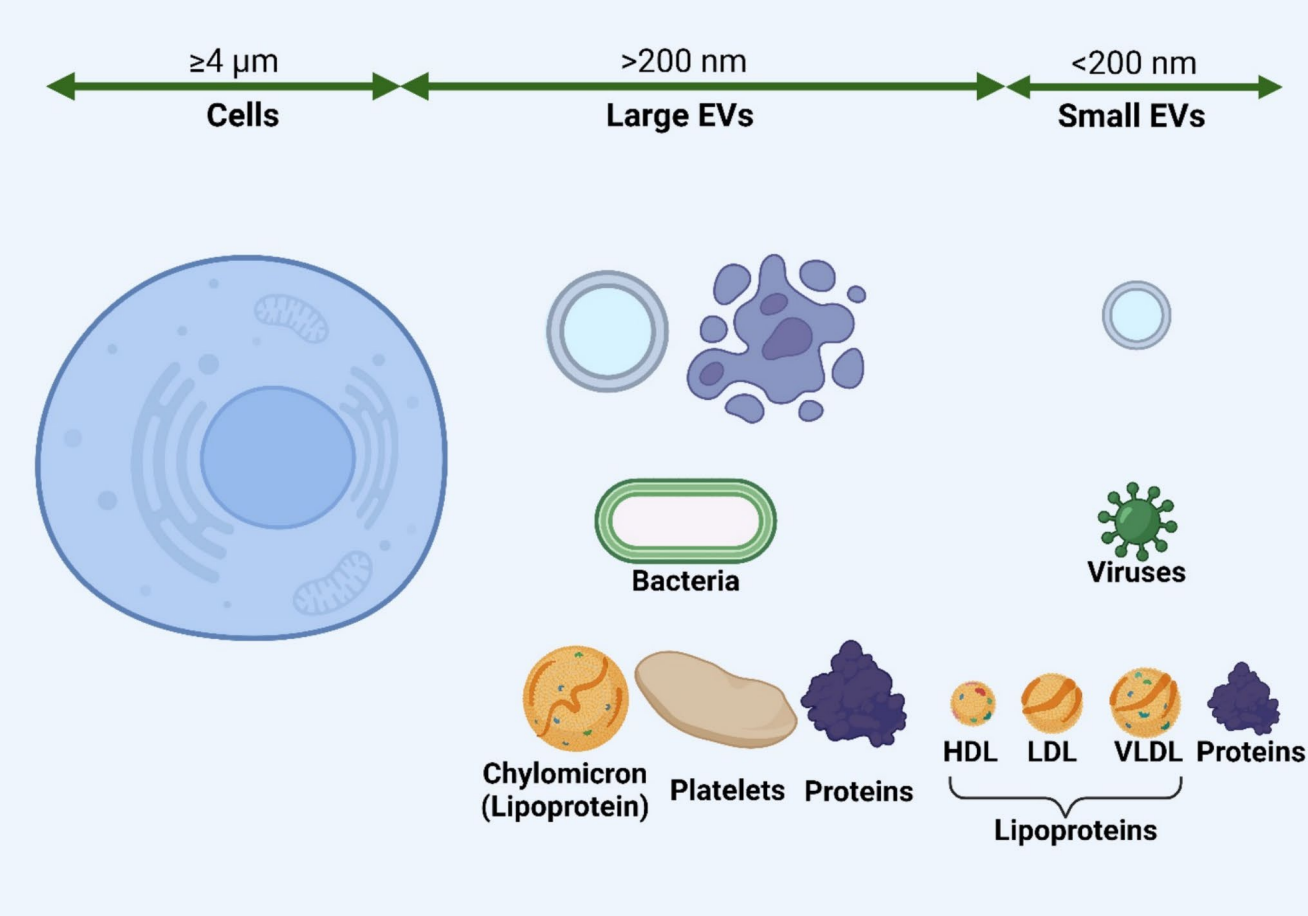
Size distribution of Extracellular vesicles (EVs). In regard to their size, small EVs (< 200 nm) overlap with viruses, protein aggregates, and lipoproteins (high density, low density, and very low-density lipoproteins). Large EVs (> 200 nm) overlap with bacteria, platelets, protein aggregates, and lipoproteins (chylomicrons). Most human cells’ sizes range from 4000 to 100,000 nm in diameter, with an average of 25,000 nm [26]

Traditionally, EVs were classified based on size and mode of biogenesis into 3 main subtypes: exosomes (30–100 nm diameter, released via endosomal multivesicular bodies); microvesicles/ectosomes (100–1000 nm, from the budding of the cell membrane); and apoptotic bodies (1000–5000 nm, released by apoptotic cells) [23]. Increasingly it is recognized that EVs are heterogeneous biological particles with significant size overlap between subtypes [24]. There is no universal subtype-specific EV marker, and demonstrating mode of biogenesis is challenging. The International Society of Extracellular Vesicles has recommended through its consensus publication, “Minimal information for studies of extracellular vesicles” (MISEV2023), to use the more generic term “EVs” [18, 25]. Alternatively, operational terms may be used, such as describing EVs based on physical properties, such as size, or biochemical composition, such as proteins [25] (Fig. 3).

EVs offer several advantages as biomarkers. Firstly, they are typically produced in greater quantities compared to biomarkers like circulating tumour cells and DNA, which are produced in particularly low quantities in patients with DTC [27]. Secondly, EV cargo is highly stable, protected by the phospholipid membrane, which prevents degradation [28]. These features underpin the growing interest in EVs for cancer biomarker development, including in TC [29].

The rapid expansion of EV research in TC has prompted this systematic review to critically evaluate and provide a comprehensive summary of all relevant preclinical studies, focusing on the diagnostic and prognostic potential of EVs, and critically appraise the methodological quality and reporting standards of included studies in accordance with MISEV2018 guidelines [18].

## Methodology

### Literature search strategy

This systematic review was performed in accordance with Preferred Reporting Items for Systematic Reviews and Meta-analyses (PRISMA) 2020 guidelines [30]. The search protocol was set a priori and registered on the Prospective Register of Systematic Reviews (PROSPERO, protocol ID CRD42022313893). Web of Science, Ovid Medline, Ovid Embase, Scopus, and Emcare were systematically searched up to 31 st March 2025 for studies published in English. Table 1 lists keywords and subject heading terms used. Reference lists of all included journal articles and relevant literature reviews were searched for additional eligible publications.

**Table 1:** Search strategy for each database used in the present systematic review List includes keywords, subject headings and their combinations, all truncations and Boleyn operators, and the number of retrieved articles from each database

Database	Keywords and subject heading terms	Number of retrieved articles
Google Scholar and PubMed	“extracellular vesicles” AND “thyroid cancer”	1320 results—limit to 200 articles to be screened further by relevance
Scopus	1 “extracellular vesicle* or exosom* or microvesicle* or microparticle* or micro-particle*” 2 thyroid* adj4 (cancer* or carcinoma* or nodule* or neoplasm* or adenoma* or tumo?r*) 1 and 2	*n* = 215
Web of Science	1 (thyroid* near/4 (cancer* or carcinoma* or nodule* or neoplasm* or adenoma* or tumo?r*) 2 (extracellular vesicle* or exosom* or microvesicle* or microparticle* or micro-particle*) 1 and 2	*n* = 91
Ovid Medline	1 exp Thyroid Neoplasms/or (thyroid* adj4 (cancer* or carcinoma* or nodule* or neoplasm* or adenoma* or tumo?r*)).tw,kf 2 exp Extracellular Vesicles/or (extracellular vesicle* or exosom* or microvesicle* or microparticle* or micro-particle*).tw,kf 1 and 2	*n* = 80
Ovid Embase	1 exp thyroid cancer/or thyroid tumor/or (thyroid* adj4 (cancer* or carcinoma* or nodule* or neoplasm* or adenoma* or tumo?r*)).tw,kw 2 exosome/or membrane vesicle/or (extracellular vesicle* or exosom* or microvesicle* or microparticle* or micro-particle*).tw,kw 1 and 2	*n* = 171

### Inclusion and exclusion criteria

This review was conducted using the population, intervention, comparison, outcomes (PICO) principle. ***Population***: TNs with a diagnosis of TC, benign, or low-risk thyroid neoplasms confirmed by histopathology. ***Intervention***: studies evaluating EVs in primary TC were included. ***Comparison:*** Control groups in studies include healthy control subjects with no thyroid disease and/or patients with benign thyroid lesions. ***Outcomes:*** The diagnostic, prognostic, predictive value of EVs and any diagnostic accuracy measures or prognostic measures were extracted.

### Screening of identified studies for eligibility

All retrieved studies were exported from all databases and uploaded to Rayyan [31], and any duplicates were removed. Two independent researchers, NA and KB, conducted a double-blinded screening of all titles and abstracts to identify potentially eligible studies. After unblinding, any conflicts in eligibility decisions were resolved through discussion between the two investigators. All included studies were subjected to full article screening to confirm eligibility in accordance with the inclusion and exclusion criteria.

### Quality assessment

All eligible studies were assessed using the Quality Assessment of Diagnostic Accuracy Studies 2 (QUADAS-2) tool for primary diagnostic accuracy studies [32]. It assesses four domains to determine the risk of bias and concerns regarding applicability: patient selection, index test (EV test), reference standard test (histopathology), patient flow, and timings of index and reference standard test. Risk of bias is determined in each domain by answering “yes,” “no,” or “unclear” to signalling questions. If the answer is “yes” to all signalling questions within the same domain, the risk of bias or concerns regarding applicability are considered low. Domains including “no” or “unclear” answers were generally considered “at risk for bias” or as having “concerns regarding applicability”

### Data extraction

The following data were extracted from included studies: first author’s name, year of publication, country of origin, thyroid cancer type and histological subtype, cancer stage, biofluid used as a source of EVs, EV isolation method, EV subpopulation studied, EV characterisation method, EV biomarkers, patients’ age and gender, sample size/number and diagnostic accuracy measures if mentioned. The authors were contacted for missing or additional information that was required.

## Results

### Included studies

**Fig. 4:**
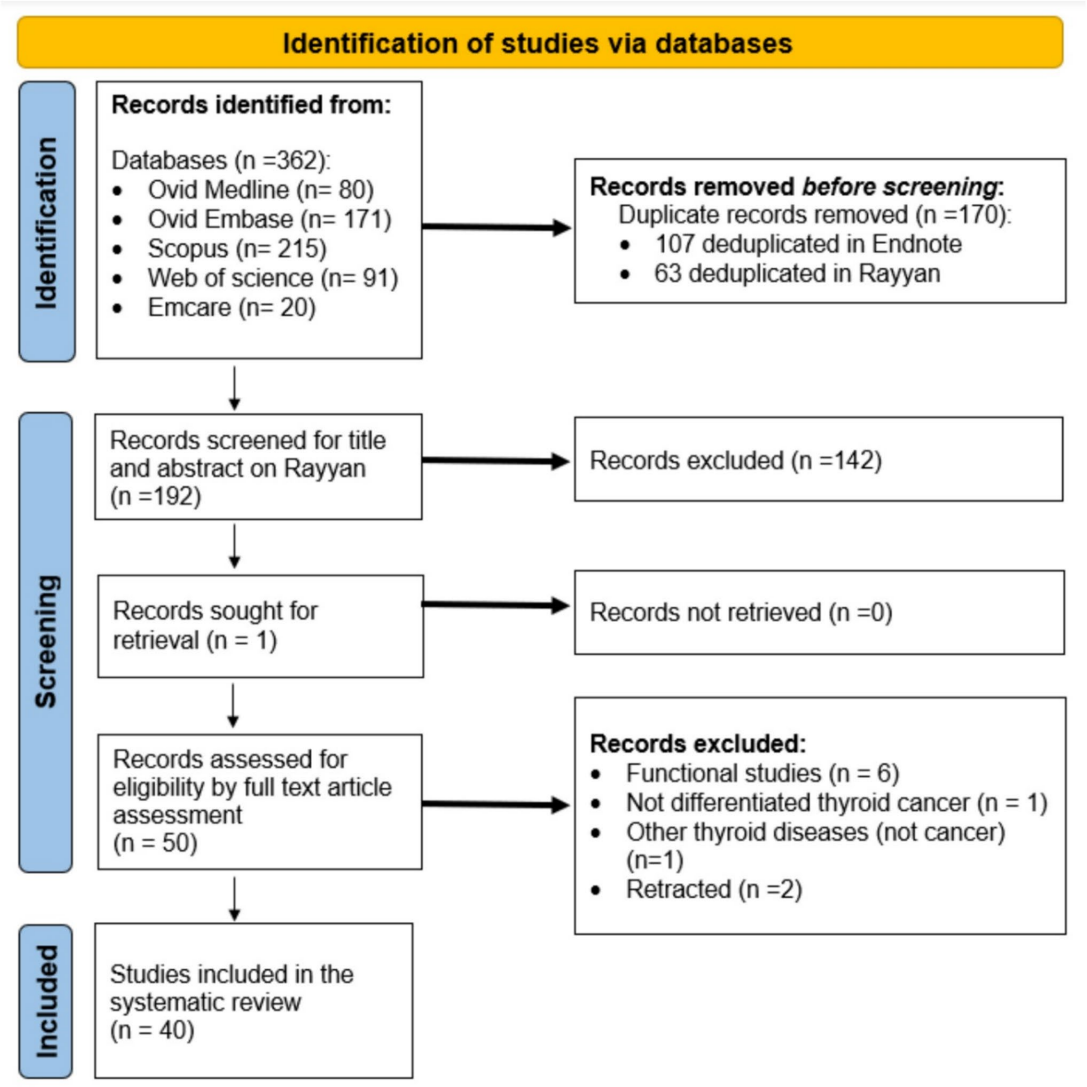
PRISMA flow chart summarising studies screening and selection procedure. Web of Science, Ovid Medline, Ovid Embase, Scopus, and Emcare were searched for relevant articles from inception to December 31^st^, 2024

A PRISMA 2020 flow diagram is shown in Fig. 4. The Literature search of the electronic databases yielded 362 studies; 192 studies remained after deduplication. Screening of titles and abstracts left 50 studies (142 studies were excluded: 12 review articles, 2 conference abstracts, and 128 irrelevant studies). The 50 remaining studies, which were related to the topic of thyroid cancer and EVs, proceeded to full-text screening. After full-text screening, 6 studies not involving patient samples or biomarkers, one studying medullary TC subtype and one studying autoimmune thyroid disease were excluded (Fig. 4). Two articles retracted from the Literature were excluded. A final number of 40 articles were included in this systematic review. Tables 2, 3, and 4 summarise their main features.

**Table 2:** Summary of studies investigating EV-derived miRNAs as thyroid cancer biomarkers

Biomarker utility (diagnostic and/or prognostic)	First author, year, (reference), country	Thyroid cancer subtype (*n*)	Control group (*n*)	Biofluid type	Biomarker quantification method	EV isolation method	miRNA studied	Main findings
Diagnostic	Boufraqech 2014 [33] USA	PTC (*n*=17)	Benign Goitre (*n*=8) HC (*n*=11)	**Serum** Thyroid vein & peripheral venous blood	**qRT-PCR** microRNA Reverse Transcription Kit (Life Technologies) Endogenous reference: miR-16	**Exosomes** Total Exosomes Isolation Kit (Life Technologies)	miR-145	**PTC v benign goitre and HC:** ↑miR-145 **Benign goitre v HC:** No significant difference
Diagnostic and prognostic	Capriglione 2022 [34] Italy	PTC-LNM (N1) (*n*=28) PTC-no LNM (N0) (*n*=30)	HC (*n*=18)	**Serum** EVs vs whole serum	**Microarray and qRT-PCR** TaqMan Advanced miRNA cDNA Synthesis Kit, miRNA Array Cards [Taqman Low-Density Arrays (TLDAs), MicroRNA Assays (Thermo Fisher Scientific, Inc.)] Endogenous reference: miR-16	**Exosomes** ExoQuick Exosome Precipitation Solution (SBI System Biosciences)	miR-127-3p miR-376a-3p miR-24-3p miR-181a-5p miR-146a-5p miR-382-5p	**PTC v HC:** ↑miR-127-3p, ↑miR-376a-3p ↓miR-24-3p, ↓miR-181a-5p, ↓miR-146a-5p, ↓miR-382-5p **PTC N1v PTC N0:** ↓miR-181a-5p, ↓miR-376a-3p, ↓miR-382-5p
Prognostic	Chen 2022 [35] China	PTC-LNM (N1) (*n*=81) PTC-no LNM (N0) (*n*=54)	-	**Plasma**	**Microarray and qRT-PCR** GeneChip miRNA 4.0 Array (Affymetrix, USA) miRCURY LNA miRNA PCR Starter and SYBR Green Kits (Qiagen, No. 339320) Endogenous reference: miR-103a-3p	**Exosomes** Microarray: ultracentrifugation qRT-PCR: exoRNeasy Serum/Plasma Midi Kit	miR-6774-3p miR-6879-5p	**PTC-N1 v PTC-N0:** ↑miR-6774-3p (AUC: 0.802; 95% CI = 0.724–0.879) ↑miR-6879-5p (AUC: 0.787; 95% CI, 0.706–0.867) The two miRNAs combined: AUC: 0.914; 95% CI, 0.865–0.962
Diagnostic	Wu 2019 [36] China	PTC-LNM (*n*=20)	Benign thyroid nodules (*n*=20)	**Serum**	**qRT-PCR** miRNeasy Micro Kit (Qiagen) cDNA synthesis kit (catalogue no. AORT-0060, Genecopoeia) Endogenous reference: miR-16-5p	**Exosomes** Ultracentrifugation	miR-21-5p	**PTC-LNM v benign thyroid nodules:** ↑miR-21-5p
Diagnostic	Zabegina 2020 [37] Russia	FTC (*n*=30)	FA (*n*=30) HC (*n*=5)	**Plasma** (EDTA) Pre-operative, after 5 days, and 4 weeks after surgery	**Microarray and qRT-PCR** miRCURY LNA miRNA Focus PCR Panels (Qiagen, USA, cat. number 339325) M-MuLV-RH RT kit (Biolabmix Ltd., Novosibirsk), FAM-labelled probe (0.2 uM) with a BioMaster HS-qPCR (2 ×) kit (Biolabmix Ltd., Novosibirsk) Endogenous reference: miR-191 or normalised to total mean of cycle thresholds of the 6 tested Let-7 family miRNAs	**Exosomes** Immunoaffinity using magnetic beads with anti-Thyroid peroxidase (TPO)	Let-7a Let-7b Let-7d Let-7f Let-7g Let-7e	**FTC v FA (in TPO (+) EVs):** ↑ Let-7b (AUC = 0.765), ↑Let-7d (AUC = 0.779), ↑Let-7f (AUC = 0.814), ↑Let-7g (AUC = 0.786), ↑Let-7e, ↑Let-7a Let-7 family miRNAs were not different between the groups in whole plasma **After thyroidectomy:** TPO (+) EV levels did not Change after 5days but decreased significantly after 4 weeks
Diagnostic	Delcorte 2022 [38] Belgium	PTC (*n*=15)	Multinodular goitre (MNG) (*n*=15) HC (*n*=10)	**Plasma** (EDTA) Pre-operative and 12–14 days post-operative EVs vs whole plasma	**qRT-PCR** M-MLV Reverse Transcriptase (Invitrogen) Takyon Probe Master Mix (Eurogentec, Belgium) Endogenous reference: Geometric mean of miR-191-5p and two spike-ins: Cel-miR-54-5p and cel-miR-34-5p	**Exosomes** Sequential iodixanol density cushion (IDC) followed by size exclusion chromatography (SEC)	miR-146b-5p miR-21a-5p	**PTC v MNG:** ↑miR-146b-5p, ↑miR-21a-5p EV miRNA levels did not change from pre-operative vs post-operative In whole plasma, miR-146b-5p and miR-21a-5p were not differentially expressed miR-146b-5p and miR-21a-5p were upregulated in PTC tissues > MNG tissues Absence of correlation between miR-146b-5p and miR-21a-5p levels in EVs and in thyroid tissue
Prognostic	Li 2022 [39] China	PTC patients with Radioactive Iodine-resistant metastases (RAIR) (*n*=48) PTC with Radioactive Iodine-avid metastases (RAI avid) (*n*=21)	-	**Plasma** -	**qRT-PCR** MiRCURY LNA miRNA PCR Starter Kit (QIAGEN, Dusseldorf, Germany) Endogenous reference: miR-103a-3p	**Exosomes** ExoReasy Serum/Serum Maxi Kit (QIAGEN, Dusseldorf, Germany)	miR-1296-5p miR-3911	**RAIR PTC v RAI avid PTC:** ↑miR-1296-5p (AUC = 0.911 with cut-off value of 2−ΔΔCT 1.9 with sensitivity 72.2% and specificity 93.6%) ↑miR-3911 (AUC = 0.728 with cut-off value of 2−ΔΔCT 1.2, sensitivity 66.7%, specificity 71.4%) Combined AUC for both miRNAs = 0.876, sensitivity, specificity of 80.0% and 85.3% EV miR-1296-5p is an independent risk factor for RAIR PTC miR-1296-5p was similarly overexpressed in the tissue of RAIR PTC > RAI avid PTC
Diagnostic	Qiao 2022 [40] China	PTC (-)	Benign thyroid nodules (-)	**Serum**	**qRT-PCR** miScript Reverse Transcription kit (Qiagen, Hilden, Germany) SYBR1 Premix Ex Taq™ II (Takara, Shiga, Japan) Endogenous reference: U6	**Exosomes** Ultracentrifugation	miR-655-3p	**PTC v benign thyroid nodules:** ↓miR-655-3p
Diagnostic	Xin 2021 [41] China	PTC (*n*=4)	Nodular goitre (*n*=4)	**Serum**	**NGS** TruSeq miRNA Sample Preparation Kit (Illumina)	**Exosomes** Exosome separation reagents (Thermo Fisher Scientific)	miR-129-2 miR-889	**PTC v nodular goitre group:** ↓miR-129-2, ↓miR-889
Diagnostic and prognostic	Wen 2021 [42] China	PTC (*n*=119)	HC (*n*=100)	**Serum** Before and 30 days and 90 days after surgery	**qRT-PCR** TaqMan MicroRNA Reverse Transcription Kit (Applied Biosystems) Synthetic spike-in reference: Cel-miR-39	**Exosomes** ExoQuick Exosome Precipitation Solution (System Biosciences)	miR-29a	**PTC v HC:** ↓miR-29a (AUC = 0.884, sensitivity = 79%, specificity = 86%) **Low EV miR-29a in PTC patients is:** an independent poor prognostic indicator for overall and recurrence free survival, correlated with larger tumour size (> 2 cm), extrathyroidal extension, and advanced TNM stage (stage III, IV vs I, II): AUC = 0.758, sensitivity = 63%, specificity = 63%), higher risk of recurrence (AUC = 0.753, sensitivity = 77%, specificity = 66%) EV miR-29a increased progressively in PTC patients’ serum 30 and 90 days after surgery
Diagnostic	Zou 2020 [43] China	PTC (*n*=100)	HC (*n*=96)	**Serum** (SST advance tubes) EVs and whole serum	**Microarray and qRT-PCR** The Exiqon-miRCURY-Ready-to-Use-PCR-Human-panel-I + II-V1.M Microarray: Exiqon, Vedbaek, Denmark) Bulge-LoopTM miRNA qRT-PCR Primer Set (RiboBio, Guangzhou, China) SYBR Green (SYBR® Premix Ex TaqTM II, TaKaRa, Dalian, China) Synthetic spike-in reference: Cel-miR-39	**Exosome** ExoQuick Exosome Precipitation Solution (System Biosciences, USA)	miR-25-3p miR-92a-3p miR-296-5p	**PTC v HCs:** ↑miR-296-5p. ↓miR-25-3p, ↓miR-92a-3p In whole serum, the three miRNAs were also significantly upregulated in PTC > HCs PTC tissues compared to healthy tissues: ↓miR-25 and miR-296 consistent with their EV levels, while miR-296-5p showed no change
Diagnostic and prognostic	Pan 2020 [44] China	PTC (*n*=13)	Nodular goitre (*n*=7)	**Plasma** (EDTA)	**NGS** NEBNext® Multiplex Small RNA library Prep Set for Illumina® (NEB, USA), Illumina Hiseq 2500 platform	**Exosomes** Sucrose density gradient ultracentrifugation	miR-5189-3p miR-24-2-5p miR-548a-3p miR-1228-5p miR-27a-3p miR-5010-3p miR-92b-3p miR-9-3p miR-598-5p miR-3161 miR-6516-5p miR-4644 miR-1283 miR-5010-3p miR-1227-3p miR-149-3p miR-210-5p miR-3662 miR-187-5p miR-5010-3p	**PTC v NG**: ↑miR-5189-3p, ↑miR-24-2-5p, ↑miR-548a-3p, ↑miR-1228-5p, ↑miR-27a-3p ↓miR-5010-3p, ↓miR-92b-3p, ↓miR-9-3p, ↓miR-4492, ↓miR-4669, ↓miR-1-3p, ↓miR-196b-5p (AUC > 0.9) Highest AUC: PTC > NG ↑miR-5189-3p (AUC = 0.951) PTC with advanced (III, IV) < early stage (I): ↓miR-5010-3p (AUC = 0.824)
Diagnostic and prognostic	Liang 2020 [45] China	PTC LNM (N1) (NGS: *n*=8, qRT-PCR: *n*=19), PTC no LNM (N0) (NGS: *n*=8, RT-PCR: *n*=16)	NGS: Nodular goitre (*n*=8) qRT-PCR: Nodular goitre (*n*=30) HC (*n*=31)	**Plasma** (EDTA)	**NGS and qRT-PCR** KAPA Library Quantification Kit and cBot Cluster Generation System using TruSeq SR Cluster Kit v3-cBot-HS (Illumia) qRT-PCR: QIAGEN miScript II RT kit and SYBR Green PCR Kit (QIAGEN) Endogenous reference: miR-30e-5p	**Exosomes** Exosome Precipitation Solution (EXOQ20A-1, SBI, Mountain View, CA, USA)	miR-16–2-3p miR-223-5p miR-34c-5p miR-101-3p miR-182-5p miR-146b-5p	**PTC v NG:** 4-miRNA panel: ↑miR-16–2-3p, ↑miR-223-5p, ↓miR-34c-5p, and ↓miR-101-3p, (AUC = 0.74 with sensitivity at 71.43%, specificity at 73.33%, cut-off < 0.9468) **NG v HC:** 3-miRNA panel: ↓miR-223-5p ↓miR-182-5p, ↓miR-146b-5p (AUC of 0.98 (sensitivity: 93.85% and specificity: 92.86%, cut-off > 0.7698). ↓miR-16–2-3p, ↓miR-223-3p, ↓miR-34c-5p **PTC LNM v no LNM:** ↑miR-182-5p, ↓miR-381-3p
Prognostic	Jiang 2020 [46] China	PTC without LNM (*n*=15), With LNM (*n*=49)	-	**Plasma** EDTA	**qRT-PCR** - Endogenous reference: U6	**Exosomes** exoRNeasy Serum/Plasma Maxi Kit	miR-21-5p miR-146b-5p miR-204-5p miR-221-3p miR-222-3p	**PTC with LNM v PTC without LNM:** ↑miR-222-3p (cut-off = 2.22: AUC = 0.834, sensitivity = 78.7%, specificity = 80.0%), ↑miR-146b-5p (AUC = 0.811, cut-off for 2−△△CT = 1.03, sensitivity = 76.6% and specificity = 86.7%), ↑miR-21-5p, ↑miR-204-5p, ↑miR-221-3p Combined panel: miR-146b-5p and miR-222-3p (AUC = 0.895, sensitivity = 85.1% and specificity = 80.0%) High miR-146b-5p and miR-222-3p are independent risk factors for LNM
Diagnostic and prognostic	Dai 2020 [47] China	PTC (NGS: *n*=17, qRT-PCR: *n*=119)	Benign thyroid nodules (NGS: *n*=10, qRT-PCR: *n*=82) HC (qRT-PCR: *n*=51)	**Serum and plasma** EDTA	**NGS and qRT-PCR** BGISEQ-500 MiR-X miRNA SYBR Kit (Takara) miDETECT A Track™ miRNA RT-qPCR Primers (Ribobio) Synthetic spike-in reference: cel-miR-39	**Exosomes** Ultracentrifugation	miR-485-3p miR-4433a-5p miR-4306 miR-376a-3p miR-204-3p miR-485-3p miR-4433a-5p miR-4306 miR-376a-3p	**PTC v BN:** ↑miR-485-3p (AUC = 0.8581; 95% CI: 0.802–0.9141; sensitivity = 85.42%, specificity = 73.33%) ↑miR-4433a-5p (AUC 0.8122; 95% CI: 0.7358–0.8886; sensitivity = 83.33%, specificity = 73.33%), ↑miR-4306, ↑miR-376a-3p, ↑miR-204-3p **PTC v HC:** ↑miR-485-3p (AUC = 0.866, 95% CI: 0.795–0.9369; sensitivity = 80.21%, and specificity = 71.19%), miR-4433a-5p (AUC = 0.8628, 95% CI: 0.791–0.9347; sensitivity = 81.25%, and specificity = 72.88%), ↑miR-4306, ↑miR-376a-3p, ↑miR-204-3p High miR-485-3p correlated with tumour size ≥ 1 cm, advanced clinical stage (AUC = 0.7532; 95% CI: 0.6494–0.8571), extrathyroidal extension (AUC = 0.7265; 95% CI: 0.6191–0.8339), BRAF mutation (AUC = 0.8903; 95% CI: 0.8255–0.9551), and lymph node metastasis (AUC = = 0.8051; 95% CI: 0.7175–0.8926) High miR-204-3p (AUC = 0.7984; 95% CI: 0.7122–0.8847) Low miR-4306 correlated with tumour size ≥ 1 cm High miR-4433a-5p was corelated with advanced clinical stage, extrathyroidal extension, BRAF mutation, and lymph node metastasis
Diagnostic	Ye 2019 [48] China	PTC (NGS: *n*=3, qRT-PCR: *n*=60)	HC (NGS: *n*=3, qRT-PCR: *n*=30)	**Serum**	**NGS and qRT-PCR** Illumina NextSeq miScript II RT Kit (Qiagen, Germany) miScript SYBR Green PCR Kit (Qiagen, Germany) Endogenous reference: miR-39	**Exosomes** Ultracentrifugation	miRNA-423-5p	**PTC v HC:** ↑miRNA-423-5p **PTC with LNM v no LNM:** ↑miRNA-423-5p
Diagnostic and prognostic	Wang 2019 [49] China	PTC: (*n*=120)	NG (*n*=29) HC (*n*=114)	**Plasma** EDTA Bulk plasma and EVs	**Microarray and qRT-PCR** Exiqon miRCURY-Ready-to-Use PCR-Human-panel-I + II-V1.M SYBR Green dye Synthetic spike-in reference: cel-miR-39 + endogenous reference: miR-16	**Exosomes** ExoQuick™ (System Biosciences, Mountain View, CL, USA)	miR-346 miR-10a-5p miR-34a-5p	**PTC v HC:** ↑miR-346, ↑miR-10a-5p, ↑miR-34a-5p (combined AUC = 0.825 (95% CI: 0.780–0.885)) **PTC v NG:** ↑miR-346, ↑miR-10a-5p, ↑miR-34a-5p (Combined AUC of three miRNAs (AUC = 0.887 (95% CI: 0.807–0.967)) The three miRNAs were upregulated in PTC patients in both whole plasma and plasma derived exosomes miR-346 and miR-34a-5p were similarly up-regulated in tumour tissues while miR-10a-5p was not different ↑miR-346 and miR-34a-5p were significantly up-regulated while miR-10a-5p was not different between PTC and matched normal tissues
Diagnostic	Samsonov 2016 [50] Russia	PTC no LNM (*n*=10), PTC LNM (*n*=10), PTC distant metastasis (*n*=14), FTC (*n*=8)	Benign Adenoma (goitre) (*n*=8)	**Plasma** EDTA Before versus after surgery	**Microarray and qRT-PCR** Cancer Focus microRNA PCR Panels ExiLENT SYBR Green master mix (both from Exiqon, Denmark) Taqman	**Exosomes** Ultracentrifugation	miR-126-3p miR-145-5p miR-31-5p miRNA-21-5p	**PTC v Benign Adenoma:** ↑miR-126-3p, ↑ miR-145-5p, ↑miR-31-5p **FTC v Benign Adenoma:** ↑miRNA-21-5p **FTC v PTC:** ↑miRNA-21-5p Levels of all 4 EV miRNAs dropped after surgery
Diagnostic and prognostic	Li 2024 [51] China	FTC (*n*=41)	FA (*n*=150)	**Plasma** EDTA Before surgery and 1 month after surgery and after radioiodine therapy	**NGS and qRT-PCR** QIAseq miRNA Library Kit 54 (Qiagen, 331505) miRCURY LNA miRNA PCR Starter Kit (Qiagen, 339320) Endogenous reference: miR-103a-3p	**Small EVs** Ultracentrifugation	miR-127-3p miR-223-5p miR-432-5p miR-146a-5p miR-151a-3p	**FTC v FA:** ↑miR-127-3p, ↑miR-223-5p, ↑miR-432-5p, ↑miR-146a-5p, ↑miR-151a-3p Combined Panel of the 5 miRNAs: AUC = 0.924, sensitivity = 0.810, specificity = 0.900 At cut-off = 0.4878, PPV = 0.895, and NPV = 0.818 The 5 miRNAs were also higher in FTC > FA tissue The 5 miRNAs in FTC were significantly correlated with increasing tumour burden, vascular invasion, distant metastasis and advanced stage disease (III, IV vs I, II) The 5 miRNAs significantly decreased postoperatively and post-radioiodine therapy
Diagnostic	Haigh 2024 [52] UK	PTC (*n*=3)	Benign thyroid tissue (*n*=3), Graves’ disease (*n*=3)	**Effluents** collected from perfused thyroid tissue biopsies maintained on tissue-on-chip technology	**NGS and qRT-PCR** QIAseq miRNA Library Kit (Qiagen) miRCURY LNA RT Kit, SYBR green miRCURY LNA PCR assays (Qiagen) Endogenous reference: miR-16-5p	**Small EVs** Ultracentrifugation	miR-375-3p miR-7-5p miR-382-5p miR-127-3p	**Graves’ disease v benign tissue derived EVs** ↑miR-375-3p, ↑miR-7-5p, ↑miR-382-5p, ↑miR-127-3p **Graves’ v PTC tissue EVs** ↑miR-375-3p, ↑ miR-7-5p **PTC v benign tissue** No significant differences
Prognostic	Gao 2024 [53] China	Thyroid cancer with BRAF V600E mutation (*n*=5), Thyroid cancer with no BRAF V600E mutation (*n*=2)	-	Thyroid cancer intraoperative irrigation solution	**Nanocavity-modulated ECL biosensor**	**Exosomes** The TransExo Cell Media Exosome precipitation Kit (Transgen biotech, China)	miRNA-222-3p	Relative positive association between miRNA-222-3p and the presence of BRAF V600E mutation
Diagnostic	D’Amico 2024 [54] Italy	PTC (*n*=7)	BG (*n*=5)	**Plasma** (EDTA) Before and 1 week after surgery	**qRT-PCR** miRCURY LNA RT Kit (QIAGEN, Hilden, Germany) miRCURY LNA SYBR® Green PCR Kit (QIAGEN, Hilden, Germany) Endogenous reference: miR-16–1-3p	**EVs** Ultracentrifugation	miR-1-3p miR-206 miR-221-3p	**PTC v BG:** ↑miR-1-3p, ↑miR-206, and ↑miR-221-3p The levels of miRNAs decreased in PTC patients down to control (BG) levels after thyroidectomy
Diagnostic	Ahmed 2024 [55], UK	FV-PTC (*n*=9) FTC (*n*=2)	FA (*n*=3) NIFTP (*n*=1) Hyperplastic TN (*n*=2)	**Plasma** (EDTA) Before surgery	**NGS** DNBSEQ-G400 platform (BGI, Hong Kong)	**L-EVs** exoRNeasy midi kit (Qiagen)	mir-195–3p mir-3176 mir-205-5p mir-208-3p mir-3529-3p let-7i-3p	**FV-PTC and FTC v FA, NIFTP, Hyperplastic TN:** ↑ mir-195–3p ↓ mir-3176, ↓mir-205-5p, ↓mir-208-3p, ↓mir-3529-3p, ↓let-7i-3p

*PTC* papillary thyroid carcinoma, *HC* healthy controls, *NGS* next generation sequencing, *qRT-PCR* quantitative real-time polymerase chain reaction, *LNM* lymph node metastasis, *HC* healthy control, *EDTA* Ethylene Diamine Tetra Acetic acid, *DTC* differentiated thyroid carcinoma, *NTA* nanoparticle tracking analysis, *DLS* dynamic light scattering, *TEM* transmission electron microscopy, *STEM* scanning-transmission electron microscopy, *WB* western blotting, *CI* confidence interval, *FV-PTC* follicular variant of *PTC*, *FA* follicular adenoma, *NIFTP* non-invasive follicular thyroid neoplasm with papillary-like nuclear features

**Table 3:** Summary of studies investigating EV-derived proteins as thyroid cancer biomarkers

Biomarker utility (diagnostic and/or prognostic)	First author, year, (reference), country	Thyroid cancer subtype (*n*)	Control group (*n*)	Biofluid type	Biomarker quantification method	EV isolation method	Proteins studied	Main findings
Diagnostic	Baicu 2017 [56], Romania	DTC (*n *= 20)	Follicular adenoma (FA) (*n *= 20) HCs (*n *= 10)	**Plasma** Before and one month after surgery	LC-MS/MS	**Microvesicles** Differential centrifugation (18,000 g for 30 min)	Cell division control protein 42 homolog (CDC42), moesin, vitronectin	**DTC and HC v FA:** ↓cell division control protein 42 homolog (CDC42) and moesin **DTC and HCs v FA:** ↑vitronectin
Diagnostic	Bavisotto 2019 [57], Italy	PTC (*n *= 13)	Benign goitre (BG) (*n *= 18)	**Plasma** EDTA Before and one week after surgery	Western blotting	**Exosomes** Ultracentrifugation (11,000 × g for 30 min)	Hsp27 Hsp60 Hsp90	**PTC v BG:** ↑Hsp27, Hsp60, and Hsp90 Hsp27, Hsp60, and Hsp90 dropped in PTC patients after surgery compared to before
Prognostic	Huang 2020 [58], Taiwan	PTC (*n *= 15) FTC (*n *= 1)		**Urine** Before, immediately after, 3 and 6 months after surgery	LC-MRM/MS	**Exosomes** EV Precipitation kit (ExoQuick-TC, SBI)	Thyroglobulin	**Before surgery: Larger PTC tumours (pT3) v smaller (pT1, 2), extra-thyroidal extension, LNM, and lymphovascular invasion** were associated with higher urinary EV Thyroglobulin **After surgery:** 4 patients showed rising EV Thyroglobulin post-surgery, although serum thyroglobulin was not detected
Prognostic	Luo 2018 [59], China	PTC with LNM (*n *= 16) PTC patients without LNM (*n *= 17)	HCs (*n *= 16)	**Serum** -	LC-MS/MS	**Exosomes** EV Precipitation (Total Exosome Isolation Reagent) and ultracentrifugation (110,000 g at 4 °C for 90 min)	CAPNS1 TLN1 SRC ALDOA ITGB2 Annexin 1 HSP27	**PTC with LNM v PTC without LNM:** ↑CAPNS1 TLN1, SRC, ALDOA and ITGB2, Annexin 1, HSP27
Diagnostic and Prognostic	Wang 2020 [60], China	Paediatric PTC (*n *= 43)	paediatric HCs (*n *= 43)	**Plasma** - Before and one week after surgery	ELISA	**Exosomes** Exosome precipitation (Total Exosome Isolation Kit)	PD‑1, PD‑L1	**Paediatric PTC v paediatric HC:** ↑PD‑1, PD‑L1 **Larger (pT3,4) v smaller (pT1,2) PTC tumours:** ↑PD-L1
Prognostic	Wang 2022 [61], Taiwan	PTC with LNM (*n *= 8), PTC with no LNM (*n *= 13)	-	**Urine** Before, 1 day, 3 and 6 months after surgery	LC-MRM/MS	**Exosomes** EV Precipitation (Exo Quick-TC, SBI)	TIMP, Angiopoietin-1	**PTC with LNM v PTC without LNM:** ↑TIMP and Angiopoietin-1
Prognostic	Wang 2024 [62], Taiwan	Post total thyroidectomy PTC (*n *= 66) and FTC (*n *= 4) patients with no evidence of recurrence	-	**Urine** 12 months after enrolment to the study, and again 1 year later	LC-MRM/MS	**Exosomes** EV Precipitation (Exo Quick-TC, SBI)	A9 peptide 13 Angiopoietin-1	**Advanced stage (IVb) v earlier stage (I, II, IVa):** was negatively correlated with A9 peptide 13 (partial r = 0.258, p = 0.008) **Microcarcinoma v larger tumours:** ↑angiopoietin 1 **Between the two intervals, no significant changes in levels of:** Thyroglobulin, Galectin-3, TKT, A8, A9 peptide 2, A9 peptide 13, Annexin-2 peptide 7, Annexin-2 peptide 16, Afamin, Angiopoietin-1, Keratin-19, TIMP peptide 5, TIMP peptide 14, Keratin-8 peptide 8, Keratin-8 peptide 17
Diagnostic and prognostic	Cao 2024 [63], China	PTMC with LNM (*n *= 10) PTMC without LNM (*n *= 10)	benign thyroid nodule (BN) (*n *= 9)	**Serum**	LC‒MS/MS and ELISA	**Small EVs** For LC‒MS/MS: SEC For ELISA: Total Exosome Separation Reagent Kit	BST2	**PTMC v BN:** 102 DEPs: ↑ 93, ↓ 9 DEPs: phagosome maturation signalling, tight junction signalling, GP6 signalling, integrin signalling, and chemokine signalling pathways. ↑BST2 (AUC = 0.906, validation cohort: AUC = 0.803, 95% CI = 0.714–0.892), RNH1(AUC = 0.878), DEFA1(AUC = 0.844), ↓ SERPINC1 (AUC = 0.867) **PTMC LNM v without LNM:** 213 DEPs: ↑ 188 and ↓ 25 DEPs: Remodelling of epithelial adherens junction signalling, integrin signalling, complement system signalling, and GP6 signalling ↑BST2
Diagnostic	Ahmed 2024 [55], UK	FV-PTC (*n *= 9) FTC (*n *= 3)	FA: (*n *= 4) NIFTP: (*n *= 3) Hyperplastic TN: (*n *= 5)	**Plasma** (EDTA) before surgery	**LC-MS/MS, Flow cytometry** **LC-MS/MS:** Bruker timsTOF Pro mass spectrometer and Evosep One liquid chromatography system (Evosep, Denmark) **Flow cytometry:** Cytoflex S (Beckman Coulter, USA)	**L-EVs** **LC-MS/MS:** differential centrifugation and SEC**, Flow cytometry:** differential centrifugation	KLK11 A1AG2 SMIM1 CXCL7 TBB1 BIP ACTN1	**FV-PTC and FTC v FA, NIFTP, Hyperplastic TN:** ↑ KLK11, A1AG2, SMIM1 ↓ CXCL7, TBB1, BIP, ACTN1

*DTC* differentiated thyroid carcinoma, *EV* extracellular vesicles, *HC* healthy controls, *LC/MS* liquid chromatography/mass spectrometry, *PTC* papillary thyroid carcinoma, *FTC* Follicular thyroid carcinoma,* LNM* lymph node metastasis, *PD-1* programmed death-1 protein, *PD-L1* programmed death-ligand 1, *ELISA* enzyme linked immunosorbent assay, *Hsp* heat shock protein, *MRM LC/MS* multiple reaction monitoring LC/MS (targeted proteomics), *PTMC* papillary thyroid microcarcinoma, *BN* benign nodules, *DEPs* differentially expressed proteins, *FV-PTC* follicular variant of PTC, *FA* follicular adenoma, *NIFTP* non-invasive follicular thyroid neoplasm with papillary-like nuclear features, *KLK11* kallikrein-related peptidase11, *A1AG2* alpha-1-acid glycoprotein 2, *SMIM1* small integral membrane protein 1, *CXCL7* chemokine (C-X-C motif) Ligand 7, *TBB1* tubulin beta chain 1, *BIP* binding immunoglobulin protein, *ACTN1* actinin alpha 1

**Table 4:** Summary of studies investigating EV-derived circRNAs, lncRNA, mRNA, DNA, procoagulant phospholipids, and biophysical characteristics as thyroid cancer biomarkers, as thyroid cancer biomarkers

Biomarker utility (diagnostic and/or prognostic)	First author, year, (reference), country	Thyroid cancer subtype (n)	Control group (n)	Biofluid type	Biomarker quantification method	EV isolation method	Biomarker studied	Main Findings
Diagnostic	Yang 2019 [64], China	PTC (n=3)	Nodular goitre (n=3)	**Serum **	**NGS and qRT-PCR** Endogenous reference: U6	**Exosomes ** Exosome isolation kit (Thermofisher Scientific, Waltham, MA, USA)	**circRNA:** circ_007293 circ_031752 circ_020135	**PTC v nodular goitre:** ↑hsa_circ_007293 and ↑hsa_circ_031752, ↓hsa_circ_020135
Diagnostic and prognostic	Lin 2021 [65], China	PTC (n=40)	HC (n=40)	**Serum **	**qRT-PCR** Endogenous reference: GAPDH	**Exosomes** ExoQuick Exosome Precipitation Solution (SBI System Biosciences)	**circRNA:** circ007293	**PTC v HC:** ↑circ007293 circ007293 higher in PTC patients with lymph node metastases and advanced tumour stage.
Diagnostic and Prognostic	Dai 2023 [66], China	PTC without Hashimoto’s thyroiditis (n=120) PTC with Hashimoto’s thyroiditis (n=44)	Benign thyroid tumours (n=60) HC (n=68)	**Serum **	**Microarray and qRT-PCR** Endogenous reference: GAPDH	**Exosomes ** Total Exosome Isolation Reagent for Serum (Life Technologies, Austin, United States)	**circRNA:** circ_0082002 circ_0003863	**PTC v benign thyroid tumours and HCs:** ↑circ_0082002, ↑circ_0003863 circ_0082002 and circ_0003863 were higher in PTC patients with higher tumour stage, lymph node metastasis, and vascular invasion.
Diagnostic	Peng 2024 [67], China	PTC patients with LNM (n=14)	HCs (n=13)	**Serum **	**NGS and qRT-PCR** Endogenous reference: β-actin	**Exosomes ** exoRNeasy Maxi Kit (Qiagen)	**circRNA:** circTACC2 circFCHO2	**PTC LNM v HC:** ↑circTACC2 ↓circFCHO2, ↓circBIRC6
Diagnostic	Dai 2020 [68], China	PTC (n=54)	HCs (n=44)	**Plasma** EDTA	qRT-PCR SYBR Green assays (TaKaRa) Endogenous reference: U6	**Exosomes** Exoquick exosome precipitation solution	**lncRNA:** DOCK9-AS2	**PTC v HC:** ↑DOCK9-AS2
Diagnostic	Feng 2024 [69] USA	DTC with LNM (n=18), DTC with no-LNM (n=20)	HCs (n=21)	**Plasma** EDTA	**qRT-PCR** PrimeDirect™ Probe RT-qPCR Mix (Takara, Japan) with β-actin primer	**Tumour-derived extracellular vesicles** Immunoaffinity capture by Click Beads bound to anti-B7H3/CD276, anti-MUC1, anti-EpCAM	**mRNA:** β-actin	**DTC v HCs:** ↑β-actin containing tumour derived EVs expressing: B7H3 (CD276) (AUC:0.91, sensitivity= 89%, specificity=81%), MUC1 (AUC:0.87, sensitivity= 95%, specificity=67%), EpCAM (AUC:0.76, sensitivity= 71%, specificity=76%) No differences in β-actin containing tumour derived EVs between DTC patients with and without LNM or among DTC patients with different T tumour stages
Prognostic	Li 2024 [70], China	PTC with LNM (n=4), without LNM (n=1)	-	**FNAC fluid **	Nanocavity modulated ECL biosensor	**Exosomes** precipitation solution from the Exosome Kit	**DNA:** BRAF V600E mutation	**PTC patients with LNM v no LNM: ** BRAF V600E mutation specific DNA was only detected in PTC patients with LNM
Prognostic	van Dreden 2009 [71], France	PTC with LNM (n=12) PTC without LNM (n=8)	HCs (n=30)	**Plasma **	Activated factor X-activated clotting time (XACT) and flow cytometry using annexin V	**Procoagulant** **microparticles** Differential centrifugation	**Procoagulant phospholipids **	**PTC with LNM v without LNM:** ↑procoagulant microparticles **PTC> HCs:** ↑procoagulant microparticles
Diagnostic and prognostic	Sun 2024 [72], China	TC with LNM (n=12) TC without LNM (n=8) 75 thyroid cancer patients (34 no-LNM, 41 with LNM)	HCs (n=25)	**Plasma**	Label-free profiling by a combination of surface-enhanced Raman spectroscopy (SERS) and deep machine learning classification algorithm by residual neural networks	**Exosomes** SEC exosome isolation columns (qEV ORIGINAL GEN 2)	**Biophysical characteristics**	**TC v HCs:** allowed discrimination with diagnostic accuracy of 96.0 % **TC with LNM N1a v TC with LNM N1b v TC without LNM:** allowed staging with diagnostic accuracy of 86.6 %

*PTC*papillary thyroid carcinoma, *HC* healthy controls, NGS: next generation sequencing, *qRT-PCR* quantitative real-time polymerase chain reaction, *LNM* lymph node metastasis, *HC* healthy control, *EDTA *Ethylene Diamine Tetra Acetic acid, *DTC* differentiated thyroid carcinoma, *TC* Thyroid cancer

### Characteristics of included studies

#### EV subtype studied

**Fig. 5:**
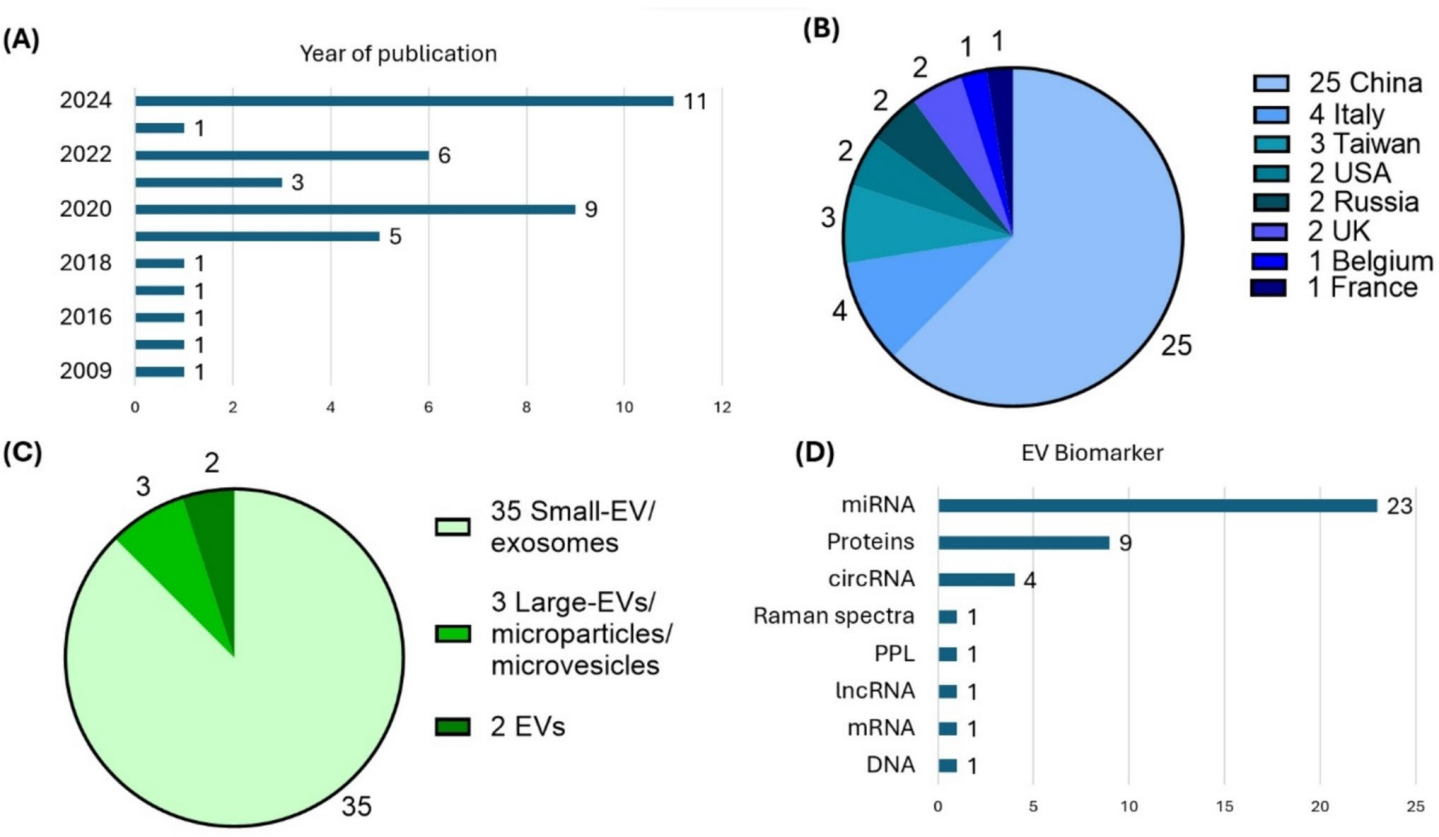
An overview of study characteristics. Studies are classified by **A** year, **B** country of the corresponding author’s affiliation, **C** EV subtype, and **D** EV biomarkers

Most studies (34/40) referred to the isolated EVs as exosomes, one used the term microparticles [71], and another used microvesicles [56]. Only three [51, 52, 63] studies used size to subtype EVs using the term “small EVs,” and one used the term “large EVs” [55]. Two studies classified EVs based on their biochemical composition by isolating and analysing EVs expressing the thyroid tissue-specific marker, thyroid peroxidase (TPO) [37] in one, and the epithelial tumour markers known to be overexpressed in thyroid cancer, namely B7H3/CD276, MUC1, and EpCAM [69] in another. Finally, two studied “EVs” without further specifying subtype [54, 69] (Fig. 5C).

#### EV biomarkers studied

Of the EV biomarkers investigated, EV RNA biomarkers were the most studied, reported in 74% (29/40) of studies, with the vast majority being microRNAs (miRNAs) (23/29), followed by circular RNA (circRNA) (4/29), then long non-coding RNAs (lncRNA) (1/29), and mRNAs (1/29). One study investigated EV DNA, while eight studied EV proteins in thyroid cancer patients. One measured the procoagulant phospholipids on EV surfaces, and finally one study applied label-free surface-enhanced Raman spectroscopy (SERS) to identify EVs’ biochemical composition and structure. No studies investigated EV lipids nor metabolites (Fig. 5D).

#### Year and country of origin

Included studies were published between 2014 and 2024, with most (77%, 30/40) published between 2020 and 2024. This shows the surge in interest for EVs as biomarkers in TC. According to corresponding authors’ affiliations, 72% (28/40) were from Asia, 64% (25/40) from China alone. No studies were conducted in Africa (Fig. 5A, B).

#### Biofluids used for EV isolation

**Fig. 6:**
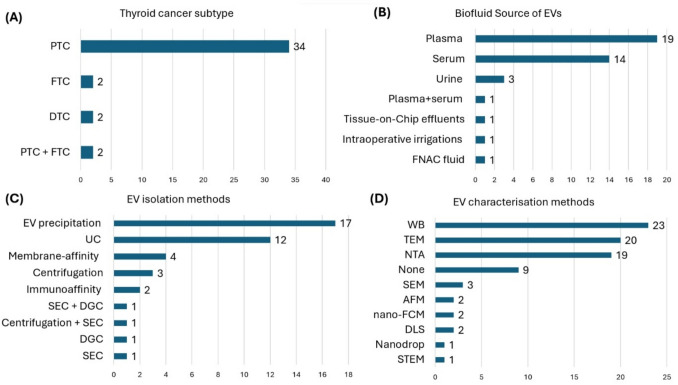
An overview of study characteristics. Studies classified by **A** thyroid cancer subtype studied **B** biofluids source used for EV isolation, **C** EV isolation methods, **D** EV characterisation technique. PTC: papillary thyroid carcinoma, FTC: follicular thyroid carcinoma, DTC: differentiated thyroid carcinoma, FNAC: fine needle aspiration cytology, DGC: density gradient ultracentrifugation, SEC: size exclusion chromatography, C: centrifugation at ~10,000 rcf/g, UC: ultracentrifugation at ~100,000 rcf/g, STEM: scanning transmission electron microscopy, DLS: dynamic light scattering, nanoFCM: AFM: atomic force microscopy; SEM: scanning electron microscopy, NTA: nanoparticle tracking analysis, TEM: transmission electron microscopy, WB: western blotting

The most used biofluid was plasma (18/40), followed by serum (14/40) and urine (3/40). One study isolated EVs from both plasma and serum. Other EV sources were effluents of thyroid tissue maintained on a Tissue-on-Chip technology (1/40); thyroid intraoperative irrigation solutions (1/40); and irrigation solution obtained from FNAC (1/40). Only 7/40 studies measured EV biomarkers in paired pre- and postoperative samples from the same patients. One study serially measured the Changes in EV biomarkers in patients who have undergone total thyroidectomy over a 2-year period. While all the other studies (31/40) performed a single pre-operative measurement (Tables 2, 3, and 4; Fig. 6B).

#### Thyroid cancer subtype and comparator groups

Most studies investigated patients with PTC (34/40). Only two investigated FTC patients, and one study included both. Two studies investigated DTC, which by definition includes PTC and FTC [3]; however, subtype data was not provided (Tables 2, 3 and 4; Fig. 6A) [56, 69]. As for control groups, 9 studies used Healthy individuals, and 11 studies used benign TN patients, while 7 studies compared PTC to both. Nine studies compared PTC patients with lymph node metastasis (LNM) to patients without. Two studies investigated EVs as biomarkers for postoperative monitoring of recurrence for DTC patients. One study compared radioactive iodine-resistant PTC patients with those having radioactive iodine-sensitive disease. Finally, one study compared PTC patients with the BRAF V600E mutation to those without. A recent prospective discovery study from our lab collected blood samples from patients with indeterminate follicular TNs, including all the range of its differential diagnoses: malignant (FV-PTC and FTC) and non-malignant TNs (hyperplastic, FA, NIFTP) [55] (Tables 2, 3 and 4).

#### Reporting of outcomes and diagnostic accuracy measures

Most studies did not report exact *p*-values and confidence intervals of measured outcomes. No studies provided power calculations. None reported complete diagnostic accuracy measures, including sensitivity, specificity, cut-off values, and area under receiver operating curve (AUC). Variable reporting of these measures precluded meta-analysis in the present systematic review.

### EV isolation techniques

**Fig. 7:**
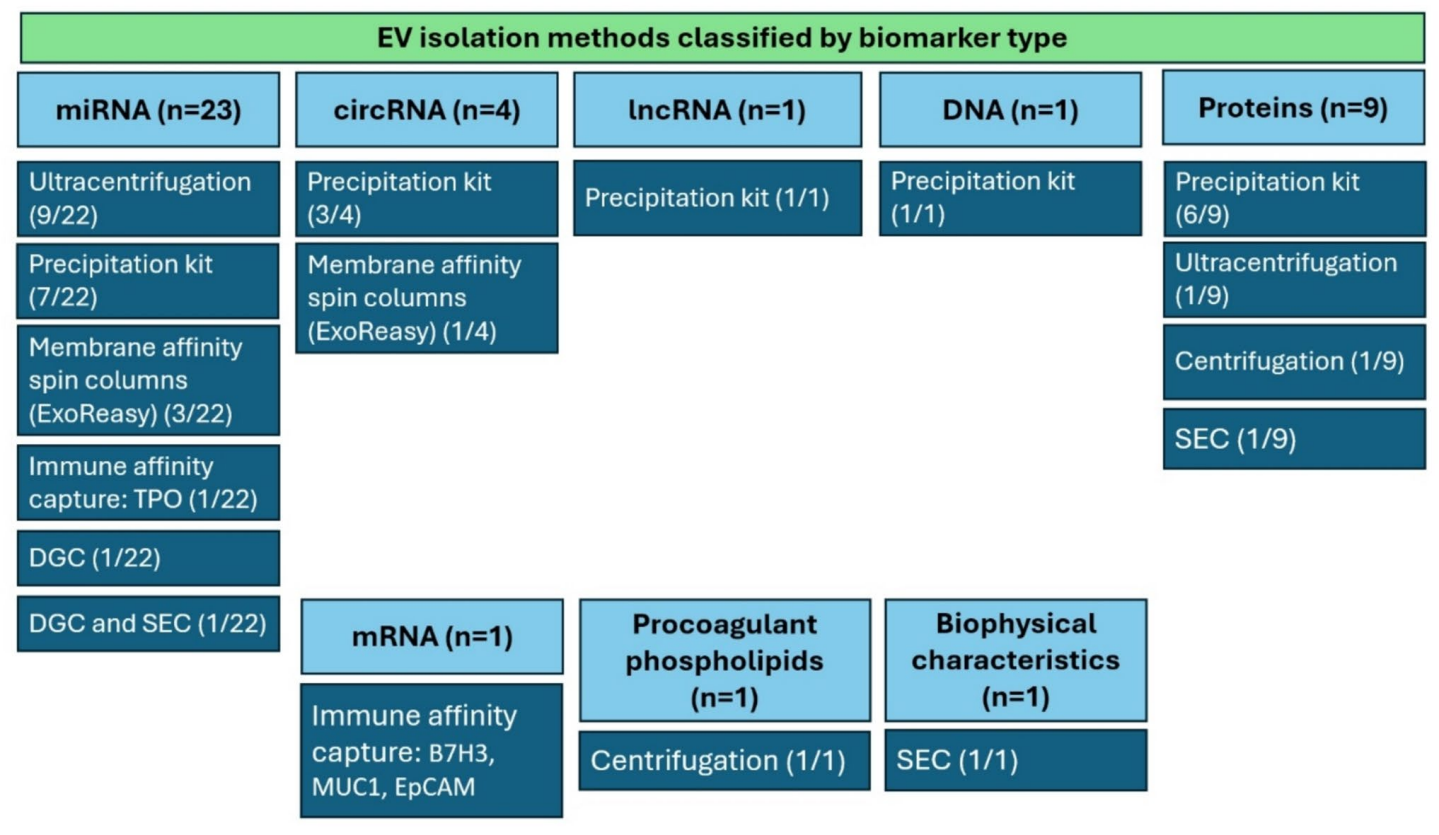
Distribution of EV isolation methods across the 40 included studies by EV biomarker type

There is no gold standard methodology for EV isolation, with no method recommended over another by MISEV2023 [18, 25]. There is considerable variation in EV isolation methods; the most used were EV precipitation kits (17/40), followed by ultracentrifugation (12/40). Other methods included differential centrifugation (2/40), membrane affinity spin columns that captured EVs based on their phospholipid membrane (2/40), size-exclusion chromatography (SEC) (1/40), and density gradient ultracentrifugation (1/40), while another study combined density gradient ultracentrifugation and SEC to achieve a higher purity EV population with minimal contaminants (1/40). Two studies used immune-affinity capture of EVs expressing specific surface proteins (2/40) and one study utilized a thyroid tissue-specific marker to isolate a subpopulation of EVs expressing thyroid peroxidase (TPO), which is a tissue-specific thyroid gland and thyroid cancer enriched protein [37, 73]. Finally, another study used three epithelial tumour markers known to be overexpressed in thyroid cancer: B7H3/CD276, MUC1, and EpCAM [69] (Figs. 6C and 7) (Tables 2, 3 and 4).

### EV characterisation techniques and adherence to MISEV2018

MISEV2018 [18] recommends characterisation of at least four EV parameters: (1) quantification of EV source (e.g., number of secreting cells, volume of biofluid, and mass of tissue); (2) EV concentrations (particle number, protein or lipid content); (3) the presence of generic EV markers; and (4) degree to which non-vesicular, co-isolated components are present. This practice permits confirmation of EVs present in the isolate and assesses co-isolated contaminants (i.e., plasma proteins or lipoproteins) [18].

Overall, most studies (30/40) used at least two different methods to characterise isolated EVs, while 9 studies assessed none of the four parameters, proceeding directly to analyse EV biomarkers. No study has fulfilled all four recommendations for EV characterisation proposed by MISEV2018 [18] (Table 5). For quantifying EV source biofluid, only 16 studies mentioned starting biofluid volume, which ranged from 100 µl to 3 ml for plasma and 200 µl to 18.5 ml for serum. Concentrations and size distribution of EVs were measured by nanoparticle tracking analysis (NTA) in 18, nanoflow cytometry in 2, dynamic Light scattering in 2, and nanodrop spectrophotometer to detect EV concentration and size distribution in one. The remaining studies did not measure EV concentrations (17/40). Generic EV positive markers were detected by western blotting in 22, including CD63, CD81, CD9, TSG101, ALG-2-interacting protein X (Alix), TSG101, flotillin-1, and β-Actin. Only 7 studies assessed co-isolated contaminant markers such as calnexin, calregulin and apolipoproteins (apoA1 and apoB). Morphology and size determination was performed by transmission electron microscopy (19/40), scanning electron microscopy (3/40) and atomic force microscopy (2/40). MISEV2018 recommends reporting an EV purity metric (e.g. protein to particle, lipid to particle, RNA to particle, or lipid to protein ratios). No included studies quantitatively estimated EV purity [18]. Additionally, MISEV2018 encourages submission of isolation and characterisation protocols to EV-TRACK [18, 74], a knowledgebase and repository for EV studies. Only two [37, 55] studies reported submitting to EV-TRACK [74] (Table 5, Fig. 6D).

**Table 5:** Adherence of the 40 included studies to MISEV2018 recommendations for EV characterisation and reporting of experimental details to EV TRACK knowledgebase

First author and reference	EV source quantification	EV abundance/concentrations	Generic EV markers	Non-EV co-isolated structures	EV purity estimation	Reporting to EV-TRACK knowledgebase	Imaging of single EVs/microscopy	Single particle analysis techniques (biophysical EV features)
Boufraqech [33]	-	-	-	-	-	-	-	-
Capriglione [34]	500 µl	-	Western blotting: CD63, TSG101, CD9	Western blotting: calregulin	-	-	-	DLS
Chen [35]	2 ml	NTA	Western blotting: CD9, TSG101, Alix	Western blotting: calnexin	-	-	TEM	NTA
Wu [36]	-	NTA	Western blotting CD63, CD81, TSG101	-	-	-	TEM	NTA
Zabegina [37]	0.5 ml	NTA	Exo-FACS Kit (CD63 and CD9)			+		NTA
Delcorte [38]	250 µl	-	ELISA: CD9, CD81 and CD63	Western blotting: ApoA1 and ApoB	-	-	TEM	
Li [39]	-	NTA	Western blotting: CD9 and TSG101, Alix	-	-	-	TEM	NTA
Qiao [40]	-	-	Western blotting: TSG101, CD63	-	-	-	-	-
Xin [41]	-	Nanodrop 2000 spectrophotometer	Western blotting: CD63, Alix, GAPDH				TEM	
Wen [42]	-	-	Western blotting: TSG101 and CD63	-	-	-	-	-
Zou [43]	200 µl	-	-	-	-	-	-	-
Pan [44]	-	NTA					TEM	NTA
Liang [45]	300 µl	NanoFCM	Western blotting: CD63 CD81, TSG101	Western blotting: calnexin	-	-	TEM	nanoFCM for sizing
Jiang [46]	-	-	-	-	-	-	-	-
Dai [47]	-	-	-	-	-	-	-	-
Ye [48]	-	NTA	Western blotting: TSG-101	-	-	-	TEM	NTA
Wang [49]	200 µl	-	-	-	-	-	-	-
Samsonov [50]	2 ml	-	Western blotting: TSG101, CD63, Alix, and β-Actin	-	-	-	AFM	Laser Diffraction analyser for sizing
Li [51]	1 ml	NTA	Western blotting: Alix, TSG101, CD81	Western blotting: calnexin	-	-	TEM	NTA
Haigh [52]	-	NTA	Western blotting: CD9, CD63 and CD81	-	-	-	-	NTA
Gao [53]	-	-	-	-	-	-	SEM	-
D'Amico [54]	4 ml	NTA	Western blotting: CD81, Alix	Western blotting: calnexin			STEM	NTA, DLS for sizing
Yang [64]	-	-	-	-	-	-	TEM	-
Lin [65]	-	NTA	Western blotting: CD63, CD81		-	-	TEM	NTA
Dai [66]	400 µl		Western Blotting: CD9, CD63, CD81		-	-	SEM	EV morphology: potentiometric analyser (Zetasizer)
Peng [67]	-	NanoFCM	Western blotting: CD63, CD81	Western blotting: calnexin	-	-	TEM	NanoFCM for size distribution
Dai [68]	-	NTA	Western blotting: CD81, CD63, TSG-101, and Alix	-	-	-	TEM	NTA
Feng [69]	100 µl	NTA	-	-	-	-	TEM. SEM	NTA
Li [70]	1 ml	-	-	-	-	-	-	-
Baicu [56]	-	-	-	-	-	-	-	-
Bavisotto [57]	3 ml	-	Western blotting: CD63 and annexin 1	-	-	-	TEM, AFM	Dynamic Light Scattering (DLS) for size distribution
Huang [58]	-	-	-	-	-	-	-	-
Luo [59]	18.5 ml pooled	-	Western blotting: CD63 and annexin 1	-	-	-	-	-
Wang [60]	-	NTA	-	-	-	-	TEM	NTA
Wang [61]	200 ml	-	-	-	-	-	-	-
Wang [62]	200 ml							
Cao [63]	500 µl	NTA	Western blotting: CD9, CD63, Alix, CD81, TSG101, flotillin-1	Western blotting: albumin, APOA1, calnexin			TEM	NTA
van Dreden [71]	500 µl	-	-	-	-	-	-	-
Sun [72]	0.4 ml	NTA	Western blotting: CD63, TSG101,	-	-	-	TEM	NTA, Raman spectroscopy
Ahmed [55]	1 ml	NTA, flow cytometry	Flow cytometry	Western blotting: ApoB	-	+	TEM	NTA, Flow cytometry

*NTA* nanoparticle tracking analysis, *DLS* dynamic light scattering, *TEM* transmission electron microscopy, *STEM* scanning-transmission electron microscopy, *WB* western blotting, *NanoFCM* nanoflow cytometry

### Assessment of risk bias

**Fig. 8:**
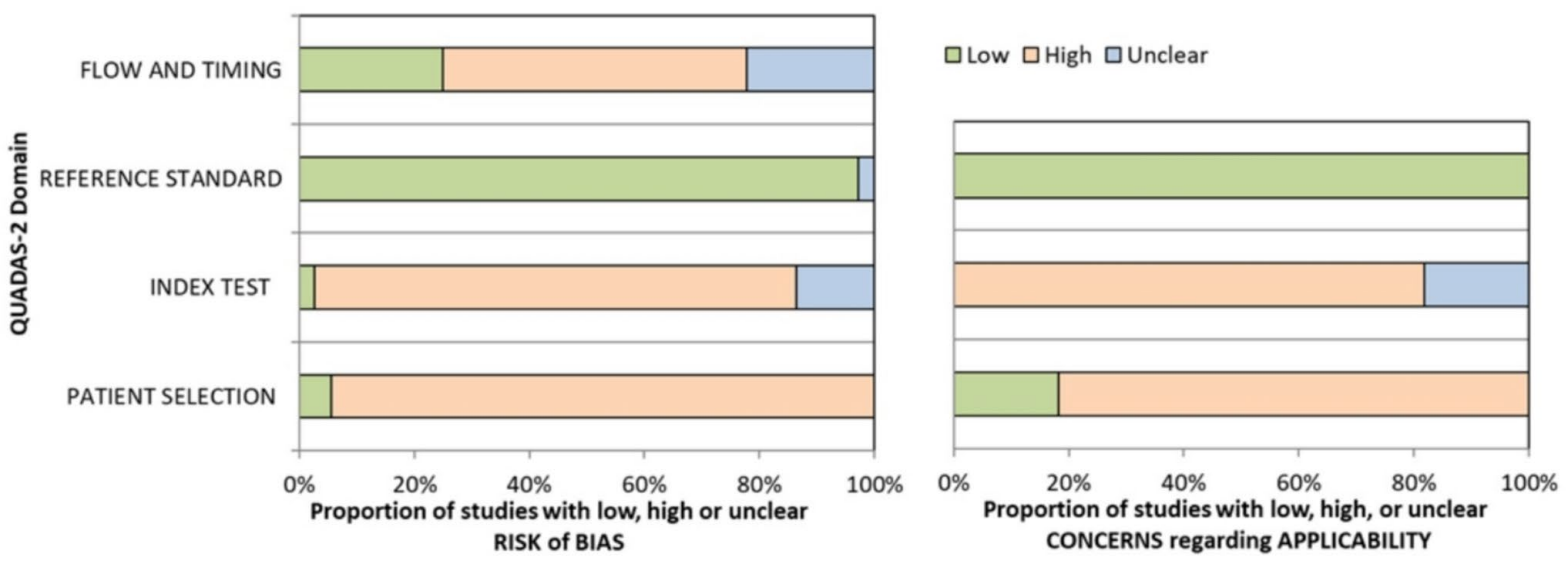
Risk of bias assessment of the 40 reviewed studies based on QUADAS 2 tool. It assesses four domains to determine the risk of bias and concerns regarding applicability: (1) patient selection, (2) index test (EV test), (3) reference standard test (histopathology), and (4) patient flow and timings of index and reference standard tests

QUADAS2 [32] risk of bias assessment all studies had an unclear risk of bias for most QUADAS2 elements (Fig. 8). Most study designs were case-control, which have an inherent risk of selection bias. Only two were prospective cohort studies, measuring EV biomarkers for postoperative surveillance of TC [58, 62]. Blinding of investigators to the reference standard test (histology) was not reported in any study; thus, risk biases in data interpretation [32].

### Thyroid cancer EV biomarker quantification techniques

#### EV RNAs

**Fig. 9:**
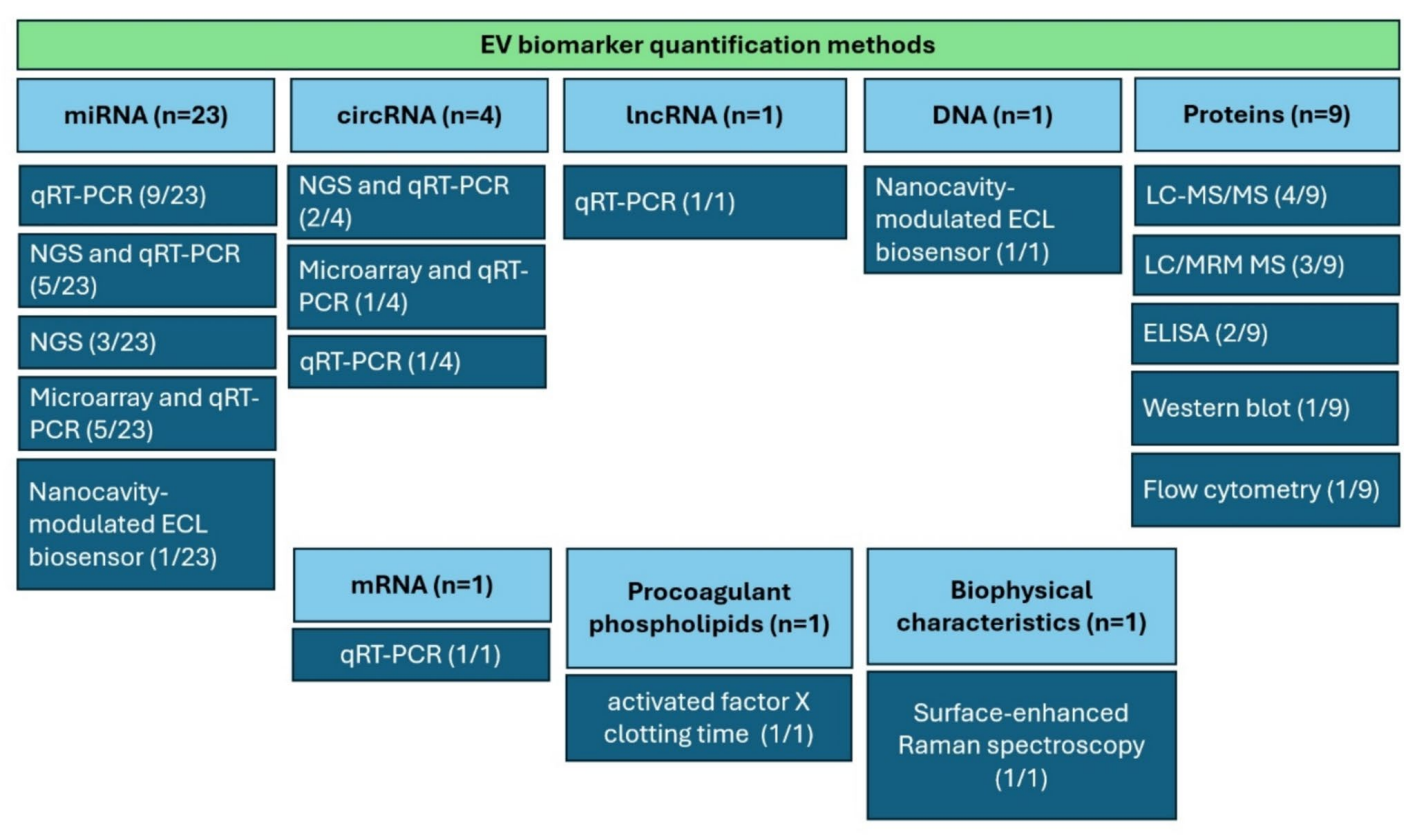
Distribution of EV biomarker quantification methods across the 40 included studies by EV biomarker type

***EV miRNAs***: The most used method was real-time quantitative polymerase chain reaction (qRT-PCR) either alone (9/22), combined with next-generation sequencing (NGS) (5/22), or microarray (5/22). NGS alone was used in two studies. One study used a nanocavity-modulated enhanced chemiluminescence (ECL) biosensor. ***EV circRNAs***: NGS and qRT-PCR were used in 2/4 studies, microarray with RT-qPCR in 1/4 and qRT-PCR alone in 1/4. ***EV lncRNA and mRNA*:** studies used qRT-PCR (Tables 2 and 4, Fig. 9).

#### EV proteins

The most used technique was proteomics via liquid chromatography mass spectrometry (LC/MS), with multiple reaction monitoring/targeted proteomics (3/8), followed by untargeted LC/MS (2/8). One study used ELISA, while another used western blotting. One study used LC/MS with ELISA validation (Table 3, Fig. 9).

#### Other EV biomarkers

EV DNA measurement was done using a nanocavity-modulated ECL biosensor. Label-free biochemical and structural properties of EVs were assessed by SERS. Procoagulant phospholipids within EV membranes were measured by the activated factor X-activated clotting time (XACT) assay (Table 4, Fig. 9).

### EV miRNA as diagnostic and prognostic markers

Nucleic acids are the most enriched molecules inside EVs, including mRNA, tRNA, rRNA, mitochondrial RNA, circRNA, lncRNA, miRNAs, and DNA [75]. MiRNAs, one of the most abundant and most studied EV cargos, are small (21–25 nucleotides) single-stranded, non-coding endogenous RNAs. They can control gene expression post-transcriptionally through base-pairing with target mRNAs, causing subsequent degradation or transient inhibition of mRNAs, inhibiting gene expression and protein production [76]. EV-enclosed miRNAs are stable, protected by the EV membrane from RNases, allowing successful transport into recipient cells. The miRNAs enclosed in EVs are segregated and concentrated from the other components of biofluids, leading to amplification of signals.

EV miRNAs are biologically active and are capable of exerting effects on cell behaviour in both physiological and pathological states, including cancer. They have been shown to promote cancer progression and metastasis [77, 78]. Thus, EV miRNAs are promising stable cancer biomarkers. Many studies have suggested the superiority of EV-miRNAs over bulk serum/plasma circulating miRNAs as diagnostic biomarkers [38, 79, 80].

#### Diagnostic potential of EV miRNAs for thyroid cancer

Comparing PTC patients to Healthy individuals, 25 miRNAs were differentially expressed: 18 and 7 miRNAs were up- and downregulated, respectively. With respect to differentially expressed miRNAs between PTC and benign thyroid nodules, a total of 47 miRNAs were significantly deregulated, while 37 and 10 miRNAs were up- and downregulated, respectively. There was an overlap between the 2 comparisons in one downregulated and ten upregulated EV miRNAs (Table 6). According to a recent meta-analysis [81], which included 10 of the 22 EV miRNA studies included in this review, the combination of several miRNA markers improved the diagnostic value and area under the curve. A Panel of 3 EV miRNAs: miR-146b-5p + miR-223-5p + miR-182-5p, gave the highest diagnostic ability to differentiate between PTC and healthy controls (AUC = 0.981, sensitivity = 93.8% (84.9–98.3), specificity equalling 92.9% (76.5–99.1 at a cut-off > 0.769)). While the combination of EV miR-346 + miR-10a-5p + miR-34a-5p demonstrated the highest performance to differentiate PTC from benign nodular goitre (AUC = 0.887, 95%CI = 0.81–0.97) [81].

**Table 6:** Summary of all differentially expressed EV-derived miRNAs in thyroid carcinoma compared to Healthy subjects and benign thyroid disease. Output identified is from 22 EV miRNA studies. The upper part of the table covers: papillary thyroid carcinoma (PTC) while end of the table covers follicular thyroid carcinoma (FTC)

Expression	PTC vs healthy control subjects	PTC vs healthy control subjects and benign thyroid nodules (overlap)	PTC vs benign thyroid nodules
**Upregulated**	miR-223-5p, miR-204-3p, miR-4306, miR-4433a-5p, miR-16-2-3p, miR-146b-5p, miR-223-3p, miR-485- 3p, miR-376a-3p, miR-423-5p, miR-25-3p, miR-296-5p, miR-10a-5p, miR-92a-3p, miR-346, miR-34a-5p, miR-127-3p, miR-145	miR-223-5p, miR-204-3p, miR-4306, miR-4433a-5p, miR-16-2-3p, miR-146b-5p, miR-223-3p, miR-485- 3p, miR-376a-3p, miR-145	miR-223-5p, miR-204-3p, miR-4306, miR-4433a-5p, miR-16-2-3p, miR-146b-5p, miR-223-3p, miR-485- 3p, miR-376a-3p, miR-889-5p, miR-3161, miR-425-5p, miR-654-5p, miR-598-5p, miR-187-3p, miR-1283, miR-4644, miR-378f, miR-7855-5p, miR-433-3p, miR-31-5p, miR-26a-5p, miR-5189-3p, miR-145-5p, miR-132-5p, miR-524-5p, miR-6516-5p, miR-219a-5p, miR-126-3p, miR-1307-3p, miR-21-5p, and miR-21a-5p, miR-145, miR-1-3p, miR-206, miR-221-3p
**Downregulated**	miR-34c-5p, miR-29a, miR-182-5p, miR-24-3p, miR-181a-5p, miR-146a-5p, and miR-382-5p	miR-34c-5p	miR-34c-5p, miR-1227-3p, miR-9-5p, miR-149-3p, miR-5010-3p, miR-187-5p, miR-101-3p, miR-3662, miR-210-5p, miR-655-3p
**FTC vs FA: upregulated—**Let-7a, Let-7b, Let-7d, Let-7f, Let-7g, Let-7e, miR-127-3p, miR-223-5p, miR-432-5p, miR-146a-5p and miR-151a-3p			
**FV-PTC and FTC vs NIFTP, FA, and hyperplastic TN: upregulated—**mir-195–3p, KLK11, A1AG2, SMIM; **downregulated—**mir-3176, mir-205-5p, mir-208-3p, mir-3529-3p, let-7i-3p, CXCL7, TBB1, BIP, ACTN1			

*PTC* papillary thyroid carcinoma, *FTC* follicular thyroid carcinoma, *FA* follicular adenoma, *FV-PTC* follicular variant of PTC, *NIFTP* non-invasive follicular thyroid neoplasm with papillary-like nuclear features, *TN* thyroid nodule, *KLK11* kallikrein-related peptidase11, *A1AG2* alpha-1-acid glycoprotein 2, *SMIM1* small integral membrane protein 1, *CXCL7* chemokine (C-X-C motif) Ligand 7, *TBB1* tubulin beta chain 1, *BIP* binding immunoglobulin protein, *ACTN1* actinin alpha 1

Upregulation of 11 miRNAs in FTC compared to FA was reported (Table 6). A Panel of 5 EV miRNAs, miR-127-3p, miR-223-5p, miR-432-5p, miR-146a-5p, and miR-151a-3p, had a combined AUC = 0.924, sensitivity of 81%, specificity of 90%, PPV of 0.895, and an NPV of 0.818 at a cut-off of 0.4878 [51]. In circulating TPO-expressing EVs, four miRNAs of the Let-7 family: Let-7f, Let-7g, Let-7d, and Let-7b, were significantly overexpressed in FTC patients with AUCs of 0.814, 0.786, 0.779, and 0.765, respectively. Interestingly, none of the Let-7 miRNAs differed in total circulating EVs (TPO positive and negative EVs) between the two patient groups [37].

One study applied tumour resection tissue on a tissue-on-chip technology perfused by EV-depleted growth medium [52]. EVs isolated from media effluents showed EV miR-375-3p, miR-7-5p, miR-382-5p, and miR-127-3p to be upregulated in EVs from Graves’ tissue versus PTC tissue or benign thyroid tissue. No significantly differentially expressed EV miRNAs were found between EVs from PTC versus benign tissue [52]. Despite the advantage of focused biomarker search to EVs originating from the thyroid lesions themselves, tissue-on-chip is an artificial environment which might change the cells’ and consequently the EVs’ molecular composition [52].

For patients with indeterminate follicular TNs, [55] an exploratory multi-platform omics approach was adopted, and candidate plasma-derived EV proteins and miRNAs that may discriminate between malignant (FV-PTC and FTC) and non-malignant nodules (hyperplastic, FA, NIFTP) were identified for future validation. In patients with malignant follicular TNs, plasma-derived large EVs had miR-195–3p upregulated, while miR-3176, miR-205−5p, miR-208−3p, miR-3529−3p, and let-7i-3p were downregulated. Furthermore, EV kallikrein-related peptidase11 (KLK11), *alpha*−1-acid glycoprotein 2 (A1AG2), and small integral membrane protein 1 (SMIM1) were upregulated, while 20 proteins were downregulated (most downregulated were chemokine (C-X-C motif) Ligand 7 (CXCL7), tubulin beta chain 1 (TBB1), binding immunoglobulin protein (BIP) and actinin alpha 1 (ACTN1)).

#### Prognostic potential of EV miRNAs for thyroid cancer

A total of 16 EV miRNAs were significantly upregulated while 15 were downregulated in PTC patients with l*ymph node metastasis* (LNM) compared to without [34, 35, 45–48]. Two EV miRNAs were up and 2 were downregulated in advanced-stage disease (III, IV) compared to early-stage disease (I, II) [44, 47, 82]. Upregulation of 2 miRNAs and the downregulation of another 2 were significantly correlated with larger tumour size [47, 82]. The upregulation of EV miR-485-3p, miR-4433a-5p and downregulation of miR-29a were correlated with locally advanced disease (extrathyroidal extension) in PTC patients [47, 82], while three EV miRNAs (miR-485-3p, miR-4433a-5p, and miRNA-222-3p) were positively correlated with the presence of BRAF V600E mutation in PTC patients [47, 53]. EV miR-1296-5p and miR-3911 were upregulated in PTC patients with radioiodine-resistant compared to radioiodine-sensitive disease. In the latter study, miR-1296-5p had an AUC = 0.911 (with a cut-off value of 2−ΔΔCT = 1.9 with sensitivity = 72.2% and specificity = 93.6%) and was found to be an independent risk factor for radioiodine resistance by multivariate regression analysis. Congruent with levels in circulating EVs of radioiodine-resistant patients, miR-1296-5p was significantly overexpressed in radioiodine-resistant PTC tumour tissue. EV miR-3911 had an AUC = 0.728 (with cut-off value of 2−ΔΔCT = 1.2 with sensitivity 66.7% and specificity 71.4%). When the 2 miRNAs were combined this gave an AUC = 0.876 [39]. In terms of survival, lower EV miR-29a in PTC patients was correlated with higher risk of recurrence and was, by multivariate analysis, an independent poor prognostic indicator of overall and recurrence-free survival [82] (Table 7).

**Table 7:** Summary of all differentially expressed EV miRNAs in TC patients with adverse prognostic factors compared to those without. The upper part of the table is concerning PTC while FTC is represented at the end of the table

Prognostic parameter	Upregulated	Downregulated
**PTC patients with lymph node metastases vs patients without**	miR-6774-3p, miR-6879-5p, miR-21-5p, miR-146b-5p, miR-204-5p, miR-221-3p, miR-222-3p, miR-485-3p, miRNA-423-5p, miR-182-5p, miR-4433a-5p, miR-26b-5p, miR-126-3p, miR-542-3p, miR-32-5p, miR-363-3p	miR-181a-5p, miR-376a-3p, miR-382-5p, miR-381-3p, miR-1912, miR-323a-5p, miR-543, miR-381-3p, miR-128-3p, miR-139-5p, miR-885-3p, miR-409-5p, miR-28-5p, miR-151a-5p, miR-490-3p
**PTC advanced TNM stage vs early stage**	miR-485-3p, miR-4433a-5p	miR-5010-3p, miR-29a
**PTC tumour size ≥ 1 cm vs < 1 cm**	miR-485-3p, miR-204-3p	miR-4306, miR-29a
**PTC extrathyroidal extension vs none**	miR-485-3p, miR-4433a-5p	miR-29a
**PTC BRAF V600E mutation vs none**	miR-485-3p, miR-4433a-5p, miRNA-222-3p	
**PTC overall survival and recurrence free survival**		miR-29a
**PTC patients with radioiodine refractory metastases vs patients with radioiodine avid metastases**	miR-1296-5p, miR-3911	
**FTC patients with high tumour burden vs low tumour burden, with vascular invasion vs without, with distant metastasis vs without, stage I, II vs III, IV**	**Upregulated**: miR-127-3p, miR-223-5p, miR-432-5p, miR-146a-5p, and miR-151a-3p	

*PTC* papillary thyroid carcinoma, *TNM* tumour-node-metastasis, *FTC* follicular thyroid carcinoma

*With respect to FTC, a* Panel of 5 EV miRNAs (miR-127-3p, miR-223-5p, miR-432-5p, miR-146a-5p, miR-151a-3p) was significantly positively correlated with increasing FTC tumour burden, vascular invasion, distant metastasis, and advanced stage disease [51] (Table 7).

#### EV miRNA levels pre- and postoperatively

Ten miRNAs were analysed preoperatively and postoperatively in PTC patients longitudinally. Eight miRNAs showed significant change in expression. The expression of miR-126-3p, miR-145-5p, miR-31-5p, miRNA-21-5p, miR-1-3p, miR-206, and miR-221-3 reduced significantly one week postoperatively [50]. In contrast, miR-29a expression progressively increased one and three months postoperatively [82]. Two miRNAs, miR-146b-5p and miR-21a-5p, remained unchanged 2 weeks postoperatively [38]. Five upregulated miRNAs in FTC patients (miR-127-3p, miR-223-5p, miR-432-5p, miR-146a-5p and miR-151a-3p) reduced significantly one month after thyroid surgery. Reduction was more marked with additional radioiodine ablation [51]. The authors concluded that these changes suggest that the EV miRNAs are more likely to be arising from the thyroid cancer itself.

#### EV miRNA levels versus serum/plasma/tissue

Plasma or serum miRNAs will include free miRNA and miRNA within EVs [38]. Studies comparing circulating EV miRNA and whole plasma/serum miRNA found 5 miRNAs that had a significant positive correlation and consistent expression levels in both (miR-24-3p, miR-296-5p, miR-10a-5p, miR-34a-5p and miR-346) [34, 43, 49]. Nine miRNAs showed no correlation between their expression levels in circulating EVs and plasma/serum, and results conflicted (miR-146b-5p, miR-21a-5p, miR-181a-5p, miR-146a-5p, and miR-382-5p, miR-127-3p, miR-376a-3p, miR-25-3p and miR-92a-3p) [34, 38, 43]. These discrepancies suggest selective miRNA packaging in EVs, indicating a complex selective sorting process that needs further research [83]. One study analysed thyroid vein blood versus peripheral blood EVs. In PTC patients, miR-145 levels were higher in thyroid venous blood. This suggests that EV miR-145 is originating from the thyroid itself [33].

Fourteen miRNAs were compared between circulating EVs and thyroid tissue. Direction of regulation was concordant for 12 miRNAs amongst both EVs and tissues [38, 39, 43, 49, 51]. In PTC patients, miR-146b-5p, miR-21a-5p, miR-346, and miR-34a-5p were upregulated, while miR-25-3p and miR-92a-3p were downregulated in both tissues and circulating EVs. In PTC patients with radioiodine-resistant metastatic disease, miR-1296-5p was upregulated in circulating EVs and tumour tissues compared to radioiodine-sensitive disease. Two miRNAs, miR-10a-5p and miR-296-5p, were upregulated in circulating EVs but not in PTC tumour tissues compared to control tissues [43, 49].

#### Concentrations of total circulating EVs in plasma

Only two papers reported concentrations of circulating EVs. In one the total circulating EVs were significantly higher in patients with indeterminate follicular TNs compared to healthy controls [55]. In the other study levels of circulating EVs showed no difference between PTC, multinodular goitre, and HCs by NTA. EV miR-146b-5p and miR-21a-5p levels, however, were significantly higher in PTC patients. This might mean that analysing EV cargo alterations might occur even at stable EV concentrations [38].

#### Heterogeneity of miRNAs reported in studies

Amongst 22 miRNA studies, only 6 miRNAs were reported in more than one as differentially expressed. MiR-21-5p was identified in 4 studies as being upregulated in thyroid cancer, while miR-146b-5p, miR-181a-5p, miR-376a-3p, miRNA-223-5p, and miRNA-423-5p were reported in 2 studies. There were no conflicting results with regards to up- or downregulation where EV miRNAs were reported in more than one study (Table 6).

#### Reference miRNAs used for normalisation of qRT-PCR

Most used was miR-16-5p in 5 studies. Only one of these 5 studies [52] demonstrated its stable expression across samples by NGS, as evidence of its suitability for EV miRNA normalisation. Other reference miRNAs used include miR-191-5p (3/22), cel-miR-39 (3/22), miR-103a-3p (3/22), U6 (2/22), miR-16-1-3p (1/22), and miR-30e-5p (1/22). It was unclear if stable expression of these reference miRNAs was confirmed prior to use (Table 2).

#### Selection of miRNAs to be investigated

Nine studies selected miRNAs to be investigated based on previous literature (Table 2). This traditional study design is hypothesis-driven, where a molecule or a group of molecules are investigated based on relevance. In contrast, there was a discovery phase in 13 studies; 8 performed miRNA NGS to identify candidates for further qRT-PCR validation, and 5 used miRNA panels to test and identify candidates for qRT-PCR validation (Table 2). Omics studies are hypothesis-generating as they provide a comprehensive, unbiased picture [14, 84], where all the samples’ molecular composition is analysed simultaneously to define a hypothesis that can then further be tested and validated [85].

### EV circRNA as diagnostic and prognostic markers

Like miRNAs, circRNAs are highly enriched and stable in EVs [86]. circRNAs (100 to > 4,000 nucleotides) have a unique single-stranded covalently closed-loop circular structure which lacks free terminal ends, conferring superior stability in tissues and fluids over linear RNAs. This serves as a crucial advantage for potential clinical application [87]. Indeed, circulating EVs subjected to harsh treatments including RNase digestion, several freeze–thaw cycles at −20 °C, and storage at 4 °C for up to 24 h had no effect on the expression levels of hsa_circ_0082002 and hsa_circ_0003863 [88]. circRNAs can regulate gene expression during transcription and translation. They can act as microRNA sponges and can even be translated into polypeptides [89]. circRNAs are involved in many pathological processes. They have been shown to play vital roles in cancer growth, angiogenesis, and metastasis [86]. Many circRNAs are implicated in TC progression with altered expression in TC tissues and blood [90].

Comparing PTC to benign thyroid tumours, 4 circRNAs (circ_007293, circ_031752, circ_0082002, and circ_0003863) were upregulated, while one (circ_020135) was downregulated. When comparing PTC patients to HCs, four circRNAs (circ007293, circ_0082002 and circ_0003863, circTACC2) were upregulated, while 2 (circFCHO2, and circBIRC6) were downregulated. In a large validation cohort of 164 PTC patients versus 60 benign thyroid tumours, hsa_circ_0082002 had an AUC = 0.848 to discriminate PTC from benign thyroid tumour patients and an AUC = 0.987 in distinguishing PTC from HCs. This high AUC indicates the potential of hsa_circ_0082002 to be a diagnostic biomarker for PTC.

Three EV circRNAs (circ_0082002, circ_0003863, and circ_007293) were significantly higher in PTC patients with more aggressive tumour features, as determined by higher stage number, LNM, and vascular invasion. The combination of 2 of these, circ_0082002 and circ_0003863, showed by logistic regression a combined AUC = 0.802 for LNM and AUC = 0.726 for vascular invasion, suggesting their potential use as prognostic biomarkers [88].

### EV lncRNA and mRNA as diagnostic and prognostic markers

LncRNA are non-protein coding RNAs longer than 200 nucleotides in length. They can regulate gene expression at multiple levels. While miRNAs can control gene regulation at the translation level by binding to mRNAs, lncRNAs can act in both mRNA dependent and independent manners. They can interact directly with DNA, RNA, and proteins, thus modulating chromatin structure and function as well as affecting RNA splicing, stability, and translation. In other words, they act as transcriptional, post-transcriptional, translational, and post-translational regulators. LncRNA play crucial roles in cancer [91], contribute to malignant phenotypical changes, and can be deregulated in many types of cancers including TC [92, 93]. Plasma EV levels of the lncRNA DOCK9-AS2 were upregulated in PTC patients compared to healthy controls and were also upregulated in PTC tumour tissues versus adjacent normal thyroid tissue [68].

In a study, concentrations of circulating tumour-derived EVs expressing B7H3/CD276, Mucin-1 (MUC1), and EpCAM in DTC compared to HCs [69], all three are epithelial tumour markers known to be overexpressed in TC [69]. Immunoaffinity was implemented using click beads with antibodies to B7H3/CD276, MUC1, and EpCAM. The click chemistry used led to irreversible immobilization of tumour-derived EVs on the beads, dramatically improving the sensitivity and specificity of immune-capture [69]. The tumour-derived EVs were then quantified by measuring their β-actin mRNA cargo levels, which were used to reflect EV abundance. Patients with DTC had significantly higher tumour-derived EV levels compared to HCs, with a high AUC of 0.91. No significant differences in tumour-derived EV levels were found between DTC patients with and without LNM or amongst DTC patients with different tumour stages [69]. Limiting the quantification of tumour-derived EVs to only those containing β-actin mRNA does not reflect the total concentrations of tumour-derived EVs but rather only those containing this particular component.

### EV DNA as diagnostic and prognostic markers

Nanocavity-modulated ECL biosensors have been developed to detect nucleic acids with exceptional sensitivity and specificity [70]. A novel biosensor composed of the luminescent Eu: CS dots and dendritic silver nanostructure/conductive gel was used to detect BRAF V600E mutation DNA levels in EVs derived from thyroid FNAC material. BRAF V600E DNA was detected in EVs of PTC patients with LNM but not in patients without LNM by the ECL biosensor. This indicates that the detection of EV BRAF V600E has the potential to predict LNM in PTC patients [70].

### EV proteins as diagnostic and prognostic markers

EVs have an abundant protein cargo, and they carry a wide range of protein types, including cell membrane-associated and cytosolic proteins [94]. These include transmembrane proteins, receptors, tetraspanins (such as CD9, CD63, and CD81), integrins, amongst others. Cytosolic proteins commonly enriched in EVs include cytoskeletal proteins, heat shock proteins, integrins, and enzymes including proteases, kinases, phosphatases, and GTPases [94]. Research has shown that EV proteins can even show post-translational modifications such as glycosylation and phosphorylation [94, 95]. While EVs share common protein compositions, they also carry proteins that are specific to cancer, enabling non-invasive cancer diagnostics [96].

Baciu et al. profiled circulating microvesicle proteins by LC/MS [56] in the plasma of DTC and FA patients and HCs and found that two proteins, cell division control protein 42 homolog (CDC42) and Moesin (MSN), both implicated in tumourigenesis in many cancers, were significantly lower in plasma microvesicles from DTC patients and HCs compared to FA patients. On the other hand, vitronectin, a secreted glycoprotein that is abundantly found in plasma and has multifunctional roles involved in cell adhesion, migration and cancer processes, was significantly higher in DTC compared to FA patients’ microvesicles, but its levels were highest in HCs [56].

Heat shock proteins (Hsps), produced in response to stress, have been implicated in carcinogenesis [97]. Hsp27, Hsp60, and Hsp90 were overexpressed in plasma exosomes of PTC compared to benign goitre patients. Postoperatively, exosome levels of these Hsps dropped significantly. These Hsps were also overexpressed in PTC tumour tissues compared to benign goitre and normal thyroid tissues, suggesting they originate from PTC tumours and are released into circulation via exosomes.

Circulating EV protein profiling of micro-PTC (PTC < 1 cm in maximum diameter) revealed 102 differentially expressed proteins: 93 upregulated and 9 downregulated compared to benign thyroid nodule patients [63]. These were enriched in signalling pathways such as phagosome maturation, tight junctions, glycoprotein VI, integrin and chemokine signalling [63].

In a study on paediatric PTC patients, circulating EV programmed cell death Ligand 1 (PD-L1) and its receptor PD-1 were significantly higher in PTC patients compared to HCs. Postoperatively, EV levels of both proteins dropped significantly in children with PTC. EV PD-L1, but not PD-1, was positively correlated with larger tumour size, suggesting increased shedding by larger tumours [60, 98]. Plasma EV PD-1 and PD-L1 could serve as diagnostic biomarkers. The PD-1/PD-L1 axis suppresses antitumour T-cell responses, aiding cancer in evading immunity. PD-L1, upregulated in most cancers, is linked to poor prognosis [99, 100]. One study found a positive correlation between plasma EV PD-L1 levels and its tumour tissue expression and lymphocyte infiltration, indicating EV PD-L1 is primarily secreted by PTC tumours [60]. EV PD-1 and PD-L1 were found to have higher diagnostic accuracy than their soluble plasma levels, likely due to biomarker concentration in EVs. Of note, EVs expressing PD-L1 are able to interact with PD-1 receptors on CD8+ T cells, leading to their inhibition, immunosuppression and tumour immune evasion [101]. Thus, EV PD-L1 might be considered a prognostic biomarker for paediatric PTC.

In patients with PTC and LNM, differentially expressed circulating EV proteins were identified using LC/MS [59]. Cytoscape pathway analysis revealed upregulation of proteins involved in integrin-mediated cell adhesion (ITGA2, ITGA2B, ITGAV, ITGB1, ITGB2, and ITGB3), their upstream (TLN1, ITGB2, SRC, and CAPNS1), and downstream proteins (TLN1, CAPNS1, and SRC) in PTC patients with LNM. This might not be surprising given that integrins play a crucial role in tumour metastasis [102]. Additionally, proteins associated with neoplasm invasiveness and metastasis (SRC, ALDOA, ACTB, MAPK1, and RAC1) and those mediating epithelial to mesenchymal transition (annexin A1, Hsp27) were upregulated in the LNM group. Cao et al. [61, 103] found that even in micro-PTC (PTC < 1 cm in maximum dimension), circulating EV protein profiles could differentiate patients with LNM from patients without, identifying 213 differentially expressed proteins (188 upregulated, 25 downregulated). These proteins were involved in epithelial-to-mesenchymal transition, integrin, epithelial adherens junction, complement system, and glycoprotein VI signalling, with bone marrow stromal cell antigen 2 (BST2 but also known as CD137) being the top upregulated protein validated by ELISA with an AUC = 0.803 (95% CI = 0.714–0.892).

Urinary EV-tissue inhibitor of metalloproteinase (TIMP) and urinary EV thyroglobulin levels were significantly higher in PTC patients with LNM using LC/MS with multiple reaction monitoring (MRM)/targeted proteomics, suggesting their potential as biomarkers for screening high-risk PTC patients for LNM [61, 103].

#### Prediction of postoperative thyroid cancer recurrence using EV proteins

Currently, serum thyroglobulin is the only blood test used to monitor PTC recurrence after ablative treatment [4]. However, anti-thyroglobulin antibodies, present in about a quarter of DTC patients, can interfere with thyroglobulin measurements [16]. To identify new biomarkers for early DTC recurrence, researchers have serially measured urinary EV peptides postoperatively [58, 103]. Urinary EV thyroglobulin was detectable in all patients, surprisingly even when not detectable in serum. In some cases, urinary EV-thyroglobulin levels at 6 months postoperatively increased, again not detectable in serum, suggesting possible recurrence but need further validation [103]. Other urinary EV peptides: galectin-3, Transketolase, A8, A9 peptide 2, A9 peptide 13, annexin-2 peptide 7, 16, afamin, angiopoietin-1, keratin-19, TIMP peptide 5,14, keratin-8 peptide 8,17 did not show increased levels, and their stability might indicate low risk of cancer recurrence [62]. Further studies with larger cohorts and longer follow-up times are needed to confirm these findings.

### EV Raman spectra and procoagulant phospholipids in diagnosis and prognosis

Surface-enhanced Raman spectroscopy (SERS) is a label-free light scattering technique that uses a monochromatic laser to illuminate a sample and detect molecule-specific vibrations, providing detailed biochemical and structural information. It identifies molecules through their characteristic Raman vibrations, such as lipids, proteins, and nucleic acids [104]. Raman spectroscopy has been used for bulk characterisation of extracellular vesicle (EV) clusters. However, single EV characterisation requires enhancement by nanoparticle substrates due to weak Raman signals from individual EVs [105]. A study used MXene-coated gold@silver core@shell nanoparticles (Au@Ag NP) to enhance EV Raman spectra. The enhanced spectra, analysed with a deep machine learning classification algorithm using residual neural networks, achieved a diagnostic accuracy of 96% in distinguishing thyroid cancer (TC) patients from healthy controls (HCs). Additionally, the model could differentiate TC patients at various lymph node metastasis (LNM) stages (N0, N1a, N1b) with an overall accuracy of 86.6% [72].

Microvesicles, which bud from the cell surface membrane, display the procoagulant phospholipid phosphatidylserine on their outer membrane. The XACT assay measures phosphatidylserine levels, with shorter clotting times indicating higher phosphatidylserine levels, reflecting the levels of phosphatidylserine exposed on the surface of EVs. In TC patients with LNM, circulating procoagulant phosphatidylserine-exposing EVs were significantly higher compared to those without LNM, and lowest in HCs. The test showed a strong correlation with phosphatidylserine-expressing EV counts via annexin V-binding and flow cytometry (*r*^2^ = 0.993). These results suggest increased phosphatidylserine-exposing EVs and a hypercoagulable state in TC patients. Thus, the XACT assay could serve as a simple, automated diagnostic and prognostic test for TC, with high consistency and reproducibility (coefficient of variation < 5%) [71].

## Discussion

To our knowledge, this is the first systematic review that focuses comprehensively on evaluating EVs as biomarkers for the diagnosis, prognosis, and monitoring of TC. We appraised the methodological quality of the included studies, with particular attention to experimental design, reporting standards, and adherence to MISEV2018 guidelines. A recent meta-analysis [81] only focused exclusively on EV miRNAs, omitting other classes of EV biomarkers, and only covered 10 of the 22 EV miRNA studies included in the present systematic review [106]. This review aims to provide an updated current overview of the published literature, highlight existing knowledge gaps, and guide future research directions.

### EV demonstrate significant diagnostic and prognostic potential for TC

A diverse spectrum of EV biomarkers was explored with good diagnostic accuracies in TC. EV miRNAs were the most studied, followed by EV protein biomarkers, while no studies investigated EV lipids or metabolites. Combining multiple EV biomarkers by logistic regression almost always improved diagnostic performance, an approach that warrants further adoption in future research [51]. EV biomarkers were correlated with multiple TC clinicopathological features, such as LNM, TNM stage, tumour size, extrathyroidal extension, pre- vs. postoperative levels, BRAF V600E mutation, radioiodine resistance, tumour recurrence, recurrence-free survival, overall survival, increasing tumour burden, vascular invasion, and distant metastasis. For example, a combined panel of two miRNAs, miR-6774-3p and miR-6879-5p, was able to discriminate PTC patients with LNM from patients without, with an AUC = 0.914 [35]. The upregulation of EV miR-1296-5p was able to discriminate patients with radioiodine resistant PTC from radioiodine sensitive, with a high AUC = 0.911, with sensitivity 72.2% and specificity 93.6% [39]. Interestingly, urinary EV thyroglobulin levels showed a rising trend in post-total thyroidectomy patients, although its serum levels remained undetectable. This finding has significant implications on early and sensitive detection of recurrence, although this requires further validation to link it to true clinical recurrence as opposed to a rise with no relevance to disease recurrence [58]. Collectively, these findings support the promise of EVs in TC diagnosis, prognosis and monitoring of recurrence with respect to specific miRNAs.

### Thyroid-tissue and tumour-specific EVs

The majority of studies measured EVs in a total pool shed from all body organs so that the population derived may not specifically be thyroid tumour-derived [107]. However, the hypothesis is that specific EV biomarkers that are consistently differentially expressed in TC patients could be used as a tumour fingerprint [108], remaining valuable (in diagnostic terms) even if not directly derived from cancer cells [52]. Cancer biomarkers may originate from cancer cells, cells in the tumour microenvironment, or as a result of the host’s immune response to cancer or other systemic processes triggered by the cancer presence [107–109]. Numerous studies have found promising EV biomarkers for cancer diagnosis, surveillance, and therapy resistance, in total EV isolates [110]. These isolates are not necessarily derived directly from the cancer cells. Further, clinically approved EV liquid biopsy-based diagnostic tests such as ExoDx Prostate IntelliScore (EPI) and ExoDx™ Lung (ALK) do not purify tissue/tumour-derived EVs. These respective tests for prostate and lung cancer quantify mRNAs extracted from total EVs as biomarkers [111, 112].

Tissue and tumour-specific markers were identified in the present systematic review. One study isolated a subpopulation of EVs expressing thyroid-tissue-specific TPO [37]. Another study isolated EVs positive for B7H3/CD276, Mucin-1 (MUC1), and EpCAM [69] for further analysis. The diagnostic value of these thyroid-tissue or tumour-derived EVs is uncertain. Given the heterogeneity of EVs in biofluids, further efforts to isolate tumour cell-specific EVs may improve future diagnostic precision [37].

### Heterogeneity in reported EV biomarkers

There was a wide variability of reported EV markers amongst studies. There are several sources of variability, including differences in TC histological subtypes, comparison groups (HCs or benign TNs), biofluid used to source EVs (plasma vs serum), and EV isolation methods. The latter is particularly heterogeneous and varied widely between studies. The most used technique was polymer precipitation, which is quick and results in high EV yields. However, EV purity is adversely affected as polymers co-precipitate EVs with water-soluble particles, including lipoproteins, proteins, and nucleic acids [113]. The second most used method was ultracentrifugation, involving high-speed centrifugation at around 100,000 g to pellet EVs [114]. Although widely used, this method is time-consuming, can damage the EVs due to the enormous centrifugal force applied, and still yields an impure EV pellet [115]. For clinical translation, EV isolation and characterisation methods should be simple, reproducible, and high throughput [116]. For instance, microfluidic-based platforms are gaining traction due to their ability to perform efficient isolation and multiplexed profiling of EVs at the single particle level, enabling clinical applications [117].

Another key source of variability was the lack of standardisation in EV miRNA qRT-PCR normalisation. No universal reference miRNA has been established to date [118]. In this review, miR-16-5p was the most used miRNA reference for normalisation of qRT-PCR. In one study [52], its stable expression across samples was demonstrated by EV miRNA NGS, suggesting its suitability for data normalisation in the context of EV miRNA biomarkers in TC. miR-16-5p has been shown in previous studies to be similarly stably expressed in EVs in many different diseases [119–121]. Used in 3 studies, miR-191-5p is an endogenous steadily expressed miRNA and has been commonly used as a reference, including for EVs [122, 123]. Used in another 3 studies, cel-miR-39 is a synthetic exogenous spike-in quality control RNA commonly applied as reference gene [124]. As a spike-in, it will only correct for experiment-related inter-sample variables and not correct for the intrinsic sample-related variability, whereas endogenous miRNA references do [125]. U6, a non-coding small nuclear RNA, was used in 2 studies. Although at least 3 times longer than mature miRNAs, it has been commonly used in the literature to normalise EV miRNA qRT-PCR [126]. It may be inherently unsuitable due to its longer length affecting its packaging into EVs [127], and not following the same miRNA regulation pathways [128]. Moreover, evidence shows varying levels of U6 in EVs from different types of cancer [129]. Heterogeneity in normalisation highlights the urgent need for future research focusing on comparing different miRNA normalisation strategies in different biofluids for TC and other diseases to identify stable reference miRNAs.

Finally, most studies selected EV biomarkers to be investigated based on previous literature. This traditional study design is hypothesis-driven, where a molecule or a group of molecules is investigated based on relevance. In contrast, there was a discovery phase in some studies where omics approaches were used. Omics studies are hypothesis-generating as they provide a comprehensive, unbiased picture, where all the samples’ molecular composition is analysed simultaneously to define a hypothesis that can then further be tested and validated [85, 130]. This approach may help streamline resources towards the most promising candidate biomarkers for future validation.

### Compliance of studies with MISEV2018 recommendations for EV characterisation

Although most studies used more than one EV characterisation method, none fulfilled MISEV2018 recommendations for EV characterisation [18]. Of the studies included, western blotting was the most applied method [112]. As a bulk analysis approach, it is, however, less helpful to analyse single or subpopulations of EVs or to understand protein localisation within EVs [131]. Morphology was assessed mainly by transmission electron microscopy. EV quantity and size were mainly determined by NTA. Collectively, these trends are consistent with previous reports, emphasising that these techniques remain most used by the EV community [114].

### Stability of EVs

Although EVs have to be isolated and stored in appropriate conditions, their cargo has been shown to be able to remarkably stable [51, 88]. Free miRNA is susceptible to degradation. Following treatment with RNase A, only half of free miRNAs were detectable, while EV miRNA and circRNA had no significant change. The protection provided by the EV membrane towards its cargo offers an advantage for clinical application over freely circulating miRNAs [51].

### Need for standardisation, adequate methodological reporting, and adherence to international guidelines

Despite encouraging findings, multiple translational challenges have been identified that need to be addressed before moving forward to clinical trials. Here, most studies suffered from incomplete reporting of many aspects of methodology and were all at risk of bias. For instance, there was an overall limited reporting of pre-analytical variables, biofluid starting volumes, how sample size was determined, missing diagnostic accuracy measures, and power calculations. The authors should strive to report pre-analytical and analytical experimental procedures using set checklists in the field like MIBlood-EV [132] for blood-based EV biomarkers and MIFlowCyt-EV [132] for EV flow cytometry experiments, and provide detailed experiment reports by submitting their experimental details to EV-TRACK [74].

In the EV field, limited reproducibility and repeatability may arise from the paucity of standardisation and adherence to international guidelines [25]. Methodological rigor, standardisation and transparency, with further larger-scale, prospective validation studies, are required to test the clinical utility of EV biomarkers for diagnosis, monitoring, and prognosis of thyroid cancer [34]. Endorsement of reporting guidelines by journals and funders could play a major role [133].

### Strengths of this systematic review

It represents the most comprehensive and up-to-date synthesis of EV biomarkers in TC to date. The search strategy was rigorous and systematically applied across five major databases, ensuring a high sensitivity for article retrieval and minimising the risk of missing relevant studies. Screening, selection, and critical appraisal of included studies were independently conducted by multiple reviewers, enhancing objectivity and reducing bias. The review was conducted with contributions from clinical and methodological experts in TC, liquid biopsy, and EV biology. This multidisciplinary collaboration ensured both the scientific accuracy and clinical relevance of the synthesis, providing valuable insights for researchers and clinicians alike.

### Limitations of the present systematic review

Despite the strengths of the present systematic literature search on EV biomarkers for thyroid cancer, our study has several limitations. Firstly, the high heterogeneity of methodologies across studies and inadequate reporting of diagnostic accuracy measures in the included studies precluded a meta-analysis. Secondly, our review and search strategy focused on DTC; however, it was noted that there were no studies investigating the role of EVs in anaplastic TC, and only one study for medullary TC [134].

The generalisability of some studies may be Limited, where 64% of included studies originated from China, potentially skewing the study populations. In China, thyroid cancer is now the third most common cancer in females compared to being the fifth worldwide [1]. Should there be geographic variations owing to differing cancer risk factors, some biomarkers may not be as applicable in differing populations. Future studies including validation in differing populations will be needed for candidate biomarkers.

### Gaps in knowledge and future directions

EV biomarker research for diagnosis, prognosis and surveillance in TC remains in its early stages. The potential to combine promising candidates into single panels should be a focus of further research as the diagnostic performance of panels generally outperformed that of individual EV markers. Most studies investigated EV biomarkers for PTC with limited focus elsewhere on FTC. Future research should aim to explore the potential of EV biomarkers in these and other less studied borderline neoplasms and TC subtypes, such as medullary and anaplastic carcinoma. Two studies compared FTC with FA. Follicular TNs, however, span a wide range of histopathologies from benign to malignant and those of yet uncertain behaviour. Any biomarker will require larger-scale prospective study to assess its real-world application. There is a clinical dilemma in identifying cancer from indeterminate follicular TNs. Only one study has addressed this [55], and although findings provide important insight in the potential of EVs to be used for diagnostic biomarkers for follicular TNs, results are still preliminary and yet require larger scale validation including all follicular TN differentials.

The following clinical challenges urgently need biomarkers Yet remain unexplored in EVs. Firstly, there is a growing trend towards de-escalating surgical treatment in TC to now offer Hemithyroidectomy as a curative option rather than total thyroidectomy for well-differentiated intrathyroidal cancers measuring 1–4 cm. Additionally, there is increasing consensus amongst oncologists to avoid radio-iodine ablation in patients with low-risk TC [13, 135]. However, a subset of patients may experience disease progression and could benefit from more extensive initial treatment if appropriately identified [136]. This underscores the need for effective non-invasive surveillance tools to support risk stratification and guide management decisions. Secondly, certain PTC subtypes, like tall cell, hobnail, diffuse sclerosing, and solid variants, have higher recurrence and metastasis rates [137]. Identifying EV biomarkers for these subtypes could improve preoperative stratification of high-risk patients and direct management. Finally, research on EVs as biomarkers of response to tyrosine kinase inhibitors in TC chemotherapy remains undiscovered.

## Conclusion

This systematic review highlights the substantial diagnostic, prognostic, and surveillance potential of EVs in TC. Their stability, biomarker-rich cargo, and their biological relevance make them promising candidates for liquid biopsy applications. However, rigorous prospective validation in large patient cohorts is required.

Standardisation of EV isolation, characterisation, and reporting, alongside adherence to established international guidelines, such as MISEV2023, is essential to ensure reproducibility and support the translation of EV biomarkers into clinical trials and routine clinical practice.

## Data Availability

All data and materials generated or analysed during this study are included in this article.
